# One-carbon metabolism during the menstrual cycle and pregnancy

**DOI:** 10.1371/journal.pcbi.1009708

**Published:** 2021-12-16

**Authors:** Ruby Kim, H. Frederik Nijhout, Michael C. Reed

**Affiliations:** 1 Department of Mathematics, Duke University, Durham, North Carolina, United States of America; 2 Department of Biology, Duke University, Durham, North Carolina, United States of America; University of Connecticut School of Medicine, UNITED STATES

## Abstract

Many enzymes in one-carbon metabolism (OCM) are up- or down-regulated by the sex hormones which vary diurnally and throughout the menstrual cycle. During pregnancy, estradiol and progesterone levels increase tremendously to modulate physiological changes in the reproductive system. In this work, we extend and improve an existing mathematical model of hepatic OCM to understand the dynamic metabolic changes that happen during the menstrual cycle and pregnancy due to estradiol variation. In particular, we add the polyamine drain on S-adenosyl methionine and the direct effects of estradiol on the enzymes cystathionine *β*-synthase (CBS), thymidylate synthase (TS), and dihydrofolate reductase (DHFR). We show that the homocysteine concentration varies inversely with estradiol concentration, discuss the fluctuations in 14 other one-carbon metabolites and velocities throughout the menstrual cycle, and draw comparisons with the literature. We then use the model to study the effects of vitamin *B*_12_, vitamin *B*_6_, and folate deficiencies and explain why homocysteine is not a good biomarker for vitamin deficiencies. Additionally, we compute homocysteine throughout pregnancy, and compare the results with experimental data. Our mathematical model explains how numerous homeostatic mechanisms in OCM function and provides new insights into how homocysteine and its deleterious effects are influenced by estradiol. The mathematical model can be used by others for further *in silico* experiments on changes in one-carbon metabolism during the menstrual cycle and pregnancy.

## Author summary

One-carbon metabolism, in which one-carbon groups are transferred between compounds, is an important part of metabolism that is found in all eukaryotic cells. It includes reactions that are crucial for cell division and the reaction that methylates DNA, the basis for epigenetics. In mammals, many enzymes of one-carbon metabolism are affected by the sex hormones testosterone and estrogen and, since estrogen varies during the menstrual cycle, one-carbon metabolites and velocities change a lot during the cycle in menstruating women. We have created a mathematical model of hepatic one-carbon metabolism to investigate these changes during the menstrual cycle and during pregnancy. The model’s predictions are consistent with the experimental and clinical literature. The model can be used to conduct *in silico* experiments to test hypotheses about the effects of possible therapeutic interventions. The model explains why women have lower homocysteine than men, which is important because homocysteine is a major biomarker for cardio-vascular disease. The model also explains why homocysteine is not a good biomarker for vitamin *B*_12_, vitamin *B*_6_, and folate deficiencies, despite claims in the literature that it is a good biomarker. The mathematical model is available and can be used by other researchers.

## Introduction

Physiological changes during the menstrual cycle and pregnancy have important health consequences, and understanding these changes is essential for holistic medical care. Approximately 5–8% of women suffer from moderate to severe premenstrual syndrome (PMS), with psychological and physical symptoms such as depressed mood, fatigue, and irritability [1]. Those experiencing premenstrual dysphoric disorder (PMDD) have symptoms that interfere with work, school, or relationships [1]. During pregnancy, unhealthy metabolic changes are linked to mother-fetal disease pathology [2]. Our goal in this paper is to study changes in an important aspect of metabolism during the menstrual cycle and pregnancy.

Many of the enzymes in one-carbon metabolism (OCM) are influenced by sex hormones, estrogen and testosterone. Estrogens activate the enzymes phosphatidylethanolamine N-methyltransferase (PEMT) [3] and cystathionine *β*-synthase (CBS) [4]. Testosterone increases the activities of Betaine-homocysteine methyltransferase (BHMT) and 5,10-methylenetetrahydrofolate reductase (MTHFR) and these activities decrease in response to estradiol in the rat liver [5]. Progesterone activates sphingomyelin synthase (SMS), which leads to higher sphingomyelin in females [6]. In this paper, we study the effects of estradiol (E2) on hepatic one-carbon metabolism. E2 is highly variable from individual to individual and, on average, menstruating women have 1–26 times more E2 than men [7–9]. In addition, E2 varies significantly throughout the menstrual cycle [7, 8, 10] and increases by about 100-fold during pregnancy [11].

**Fig 1:**
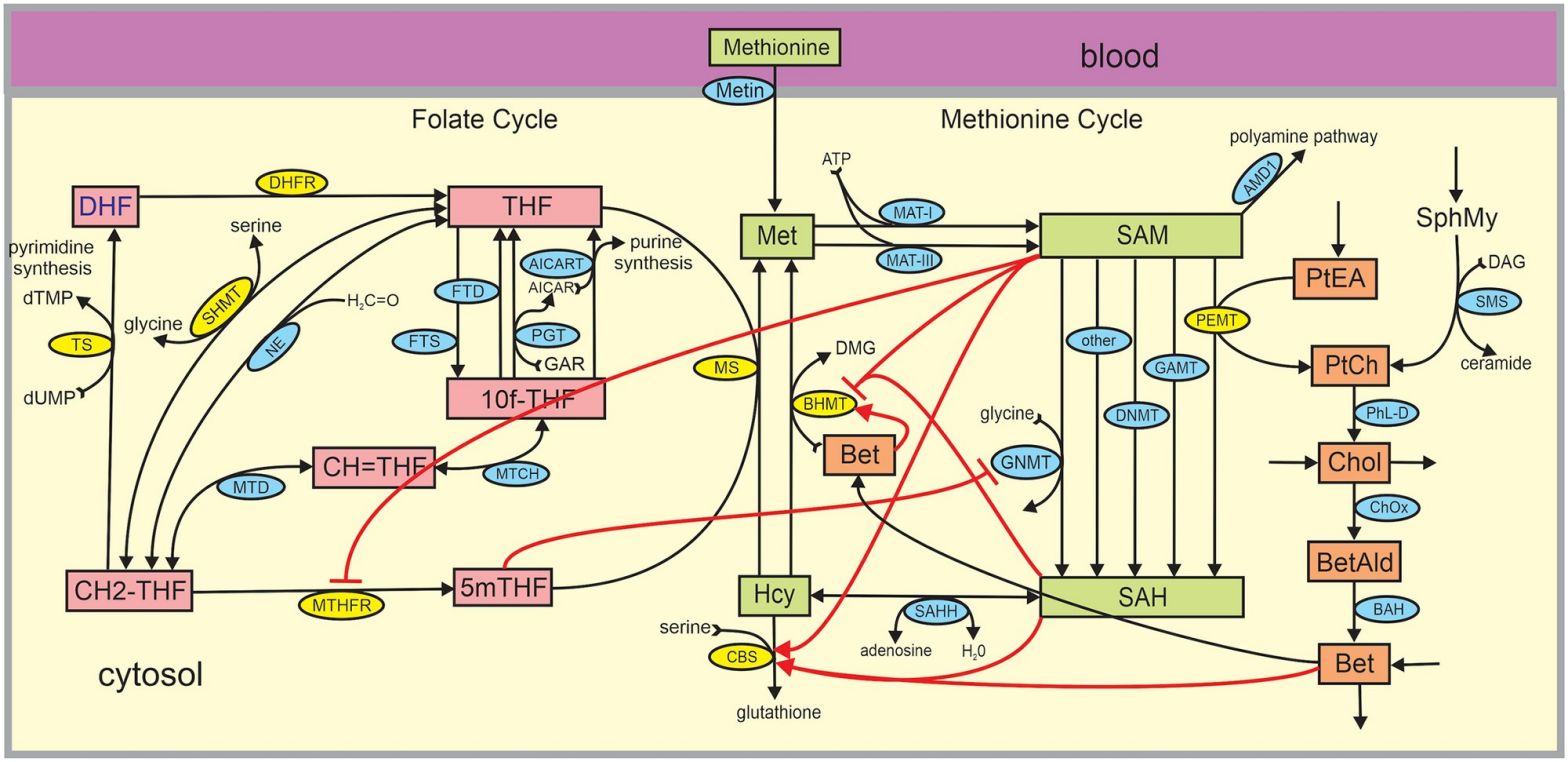
One-carbon metabolism. Substrates are indicated by rectangular boxes, green in the methionine cycle and red in the folate cycle. Each black arrow represents a biochemical reaction and the blue and yellow ellipses on the arrows contain the acronyms of the enzymes that catalyze the reactions. The yellow ellipses indicate the enzymes that are up- or down-regulated in females (see Table 1). Each red arrow is a long-range allosteric influence, either activation (arrow) or inhibition (bar). Substrate abbreviations: Met, methionine; SAM, S-adenosylmethionine; SAH, S-adenosylhomocysteine; Hcy, homocysteine; 5mTHF, 5-methyltetrahydrofolate; THF, tetrahydrofolate; 10fTHF, 10-formyltetrahydrofolate; DHF, dihydrofolate; CH2-THF, 5,10-methylenetrahydrofolate; CH = THF, 5,10-methenyltetrahydrofolate; SphMY, sphingomyelin; PtEA, phosphotidylethanolamine; Cho, choline; Bet-Ald, betaine aldehyde; Bet, betaine. Enzyme abbreviations: AICAR(T), aminoimidazolecarboxamide ribonucleotide (transferase); FTD, 10-formyltetrahydrofolate dehydrogenase; FTS, 10-formyltetrahydrofolate synthase; MTCH, 5,10-methylenetetrahydrofolate cyclohydrolase; MTD, 5,10-methylenetetrahydrofolate dehydrogenase; MTHFR, 5,10-methylenetetrahydrofolate reductase; TS, thymidylate synthase; SHMT, serine hydroxymethyltransferase; PGT, phosphoribosyl glycinamidetransformalase; DHFR, dihydrofolate reductase; NE, nonenzymatic interconversion of THF and 5,10-CH2-THF; MAT-I,methionine adenosyl transferase I; MAT-III, methionine adenosyl transferase III; AMD1, adenosylmethionine decarboxylase; GNMT, glycine N-methyltransferase; AS3MT, arsenic methyltransferase; PEMT, phosphotidylethanolamine methyltransferase; GAMT, gunadino-acetate methyltransferase; DNMT, DNA-methyltransferase; SAHH, S-adenosylhomocysteine hydrolase; CBS, cystathionine *β*-synthase; MS, methionine synthase; SMS, sphingomyelin synthase; PhL-D, phospholipade D; ChOx, choline oxidase; BAH, betaine aldehyde dehydrogenase; BHMT, betaine-homocysteine methyltransferase.

Two authors of this paper (MCR and HFN) created, with colleagues, a mathematical model to study steady state sex differences in one-carbon metabolism in 2018 [12]. In this paper, we use additional information to extend the model and use it to study changes in OCM during the menstrual cycle and during pregnancy as well as to present further steady-state results on vitamin deficiencies. A schematic diagram is provided in Fig 1. The yellow ellipses indicate enzymes that are up- or down- regulated on average in menstruating women, compared to men. Details of the differential equations are provided in [12]; here we indicate the largest changes to the 2018 model (see Methods). One result of the 2018 paper was the explanation for why menstruating women have lower homocysteine (Hcy) than men on average. Because of the upregulation of PEMT, menstruating women have higher betaine, which activates CBS and lowers Hcy [12]. More recently, experimentalists have also observed that E2 directly stimulates the transcription of CBS [4]. Furthermore, E2 also stimulates the enzymes thymidylate synthase (TS) and dihydrofolate reductase (DHFR) which are involved in the folate cycle [13, 14]. We have modified the mathematical model [12] to include these effects and have also added the enzyme adenosylmethionine decarboxylase (AMD1), which drains mass from S-adenosylmethionine (SAM) for the polyamine pathway. The polyamine mechanism was included because it drains mass in the methionine cycle and therefore the remethylation flux from Hcy to methionine (Met) does not all return to Hcy; this affects the magnitude of changes under Vitamin *B*_12_ deprivation. All other changes to the model in [12] are minor and do not change model results. The major changes to the model are described in the Methods, and further discussed in S1 Text. The complete MATLAB code is provided in S2 Text.

No mathematical model can represent the underlying physiology perfectly. We make a number of simplifications. For example, serine is held constant, we make a very simple model of the synthesis of glutathione, and we are assuming that all the folates are in monoglutamyl form. This is because in this paper we are concentrating on the effects of estrogen on OCM. In addition, this is a model of OCM metabolism in the liver and we are assuming that Hcy in the liver is proportional to Hcy in the plasma (where it is usually measured). Finally, we note that enzyme concentrations in the liver differ by about 25% from individual to individual [15–17]. Therefore, the specific concentrations and velocities depicted in Fig 1 will vary from individual to individual. Our goal is to investigate the system properties of OCM and the many regulatory mechanisms that create those properties.

In the section “Estrogen and homocysteine” we discuss how estradiol variation during an idealized 28-day menstrual cycle in the mathematical model drives 28-day variation in Hcy. Our model predicts that women using oral contraceptives will have an elevated average Hcy, and this is consistent with experimental findings [18, 19]. In the section “Fluctuations in OCM during the menstrual cycle” we show how other metabolites and velocities in the folate and methionine cycles vary throughout the menstrual cycle, and we connect these results with the literature. In the section “Is homocysteine a good biomarker for vitamin deficiencies?” we explore vitamin *B*_12_, vitamin *B*_6_, and folate deficiencies in the mathematical model for the “average” man versus the “average” woman, and explain why Hcy is not a good biomarker for vitamin deficiencies, despite claims in the literature. And finally, in the section “Homocysteine in pregnancy” we compute the effects of E2 on lowering Hcy throughout pregnancy and compare with experimental data.

## Methods

The schematic diagram of the mathematical model is shown in Fig 1. The pink boxes in the folate cycle and the green boxes in the methionine cycle and the orange boxes in the choline pathway indicate metabolites whose concentrations can change in the mathematical model (variable metabolites). The arrows represent biochemical reactions and the blue and yellow ellipses show the acronyms of the enzymes catalyzing the reactions. The yellow ellipses indicate the enzymes that are substantially up- or down-regulated in females. Full names of the enzymes and substrates are in the legend of Fig 1. The allosteric interactions crucial for our investigations in this study are indicated by red arrows. There are other allosteric interactions in the model that are not included in Fig 1. For example, S-adenosyl-homocysteine (SAH) inhibits each of the methyltransferases and SAM affects both of the enzymes that synthesize it from Met. This mathematical model is an extension of the model used in [12], so we describe only what has been added or revised. The complete mathematical model is described in S1 Text.

### Cystathionine *β*-synthase (CBS)

The kinetics of CBS are standard Michaelis-Menten with *K*_*m*_ = 1000*μ*M for Hcy taken from [20]. The second term is the activation of CBS by SAM and SAH. The form of the activation was derived by non-linear regression on the data in [21] and scaled so that it equals 1 when the system is at the normal male steady state. The third term is the activation of CBS by betaine using the data in [22] as described in [12].

$$\begin{array}{cc} \; & V_{\text{CBS}}\left(\left[Hcy\right],\left[SAM\right],\left[SAH\right],\left[Bet\right]\right)\\ & \;\;=(\frac{h\left(E2\right)V_{max}\left[Hcy\right]}{K_{m}+\left[Hcy\right]})(\frac{4.6([SAM]+[SAH])}{14+([SAM]+[SAH])})\\ & \;\;\;\cdot (1+H\left(\left[Bet\right]-315\right)\cdot \frac{0.25([Bet]-315)}{100+([Bet]-315)}), \end{array}$$

where H([Bet]-315) is the Heaviside function which is equal to zero when [Bet] is at or below steady state (315) and equals one otherwise. The third term equals 1 for males and rises to 1.25 for females. The factor *h*(*E*2) is the direct effect of estradiol on CBS activity (see below). The CBS reaction utilizes serine, but we assume that the serine concentration is constant and so it is part of *V*_*max*_ = 880*μ*M.

### Glutathione

Glutathione (GSH), a tripeptide consisting of cysteine, glycine, and glutamate, is the major cellular anti-oxidant [23]. It is synthesized by the transsulfuration pathway: Hcy is converted to cystathione which is converted to cysteine to which glycine and glutamate are added. A large, detailed mathematical model of OCM including the transsulfuration pathway and the mitochondria was created by two of the authors and colleagues [24]. For the purposes of this paper, we create GSH by a simple linear reaction from Hcy and choose the degradation rate of GSH to be such that the steady state concentration of GSH is approximately 6500 *μ*M as in [24].

### Polyamine pathway

The drain from SAM to the polyamine pathway is known to make up a significant amount of flux from the methionine cycle [25, 26]. The enzyme AMD1 catalyzes the conversion of SAM to dcSAM, an important precursor for polyamine synthesis; see Fig 1. The kinetics of AMD1 in this mathematical model are Michaelis Menten with *K*_*m*_ = 245*μ*M for SAM taken from [27]. There is not much reliable information on the size of the polyamine flux relative to the flux around the methionine cycle. We chose the *V*_*max*_ so that the polyamine flux is around 20% of the flux from Met to SAM.

$$\begin{array}{c} V_{AMD1}\left(\left[SAM\right]\right)=\frac{V_{max}\left[SAM\right]}{K_{m}+\left[SAM\right]} \end{array}$$ (1)

The differential equation for SAM from the previous model [12] was modified to include the polyamine drain modeled by Eq (1).

### Direct effects of estradiol on PEMT and CBS

Estradiol stimulates PEMT enzyme activity in human hepatocytes [3]. We used a Michaelis-Menten form in Eq (2) for PEMT activity fold, *g*(*E*2), dependent on estradiol concentration, *E*2, with *α*_1_, *α*_2_ > 0. *g*(*E*2) multiplies the *V*_*max*_ of PEMT, and increases with estradiol.

$$\begin{array}{c} g\left(E2\right)=\frac{\alpha_{1}E2}{\alpha_{2}+E2}+1 \end{array}$$ (2)

In Eq (3), the PEMT fold equals 1 when *E*2 = 0, and approaches 1 + *α*_1_ as *E*2 increases. The parameters *α*_1_ = 1.29 and *α*_2_ = 1.04 were determined using Matlab’s nonlinear regression function *nlinfit* with data from [3].

The 2018 paper [12] included sex differences in the enzymes PEMT, BHMT, SHMT, MS, and MTHFR. There is recent evidence of sex differences in CBS as well. In particular, CBS is transcriptionally upregulated by estradiol [4, 28]. We used a Michaelis-Menten form in Eq (3) to model CBS activity fold, *h*(*E*2), dependent on estradiol concentration, *E*2, with *β*_1_, *β*_2_ > 0. *h*(*E*2) is multiplied to the *V*_*max*_ of CBS, and increases with estradiol.

$$\begin{array}{c} h\left(E2\right)=\frac{\beta_{1}\left(E2-.09\right)}{\beta_{2}+\left(E2-.09\right)}+1 \end{array}$$ (3)

In Eq (3), the CBS fold increases up to 1 + *β*_1_, and the parameters *β*_1_ = 1 and *β*_2_ = 0.67 were chosen so that CBS activity increases 2-fold during pregnancy [28] and the female-male ratio of CBS activity is 5.1/3.8 [29]. For males, the model estradiol concentration is 0.09 nanomolar (nM) [9] and *h*(*E*2) = 1.

### Effects of estradiol on TS and DHFR

**Fig 2:**
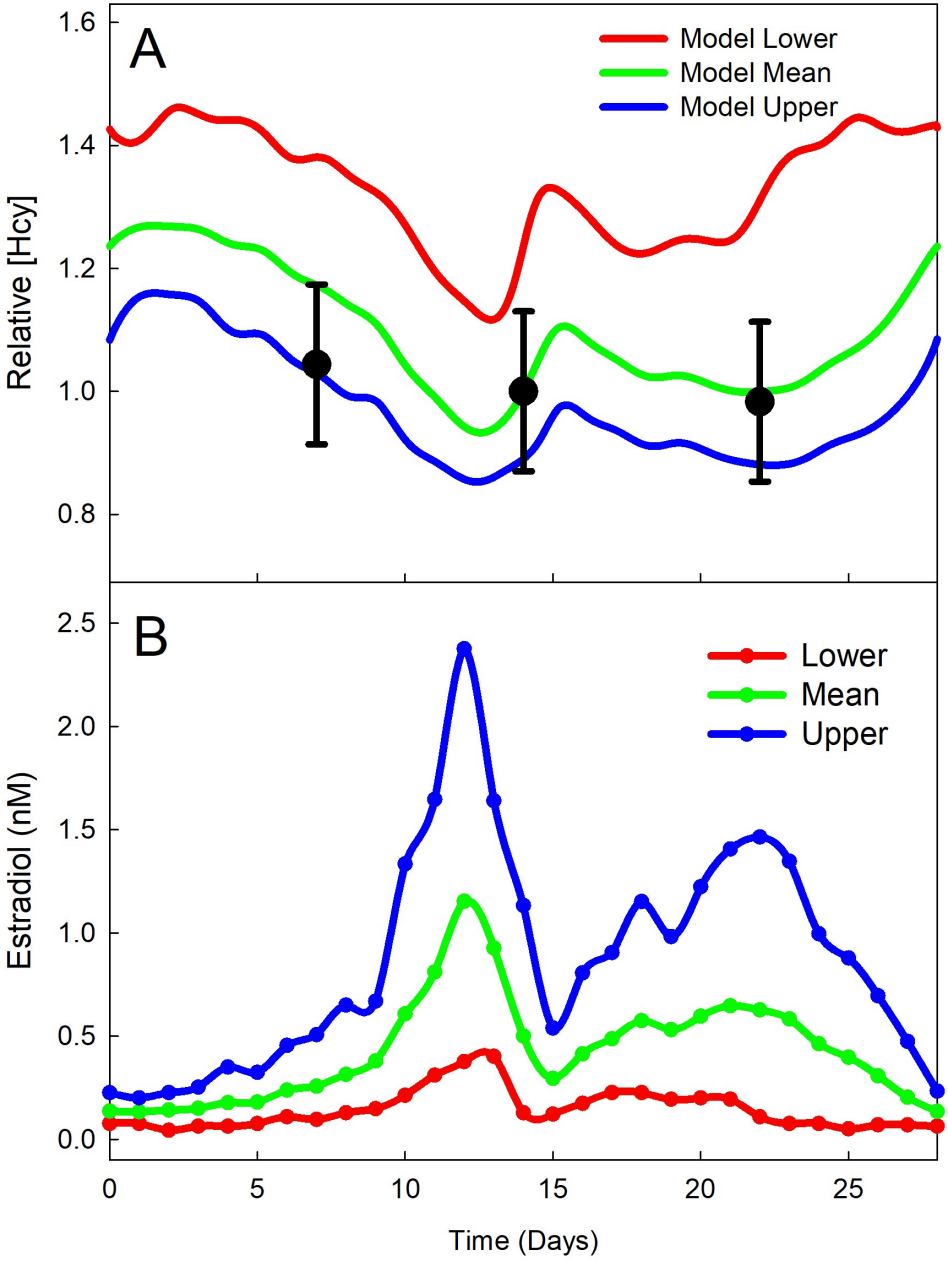
Hcy and E2 throughout the menstrual cycle. Model predictions of relative Hcy variation using three different estradiol curves are plotted. (Panel A). The Hcy experimental data and the Hcy model curves are plotted relative to the Hcy concentration on day 14 (predicted ovulation) of the mean estradiol curve. The Hcy data [44] is plotted with error bars representing the standard deviation. The model estradiol curves were determined by interpolation of data from [7, 42, 43]. The data is regraphed in Panel B, where the three curves are mean estradiol (Mean), the 95th percentile (Upper), and the 5th percentile (Lower). The errors bars are standard deviation. The corresponding model Hcy curve for each estradiol curve is plotted in Panel A in the same color with the same label.

Experiments suggest that estradiol also directly stimulates thymidylate synthase (TS) and dihydrofolate reductase (DHFR) [13, 14]. Xie et al. [13] found that a treatment of 10 nM E2 in MCF-7 human breast cancer cells increased TS activity by 30–100%. In menstruating women, the average peak E2 concentration is 1 nM (see Fig 2). We assume that 1 nM E2 increases TS activity by $\frac{1}{10}\cdot 100\%=10\%$. Aitken et al. [14] showed that E2 increases TS and DHFR activity. The function *u*(*E*2) models both TS and DHFR activity dependent on E2.

$$\begin{array}{c} u\left(E2\right)=\frac{a_{1}E2}{a_{2}+E2}+1 \end{array}$$ (4)

where *a*_1_ = 2 and *a*_2_ = 19. In the model, TS and DHFR activity increase 1.1-fold at 1 nM E2, and are bounded by a 3-fold increase for large E2. This function can easily be adjusted for each of TS and DHFR when more data become available.

### Estradiol dynamics

The model in this paper predicts the effects of dynamic estradiol on OCM in two cases: throughout the menstrual cycle and during pregnancy. Here, we describe how we modeled estradiol variation in each case.

Estradiol levels vary significantly throughout the menstrual cycle, inducing rhythmic changes in OCM metabolites over an approximately 28-day period [8, 10]. Estradiol concentrations on each day of the menstrual cycle were measured in [7], where the 95th percentile, the 5th percentile, and the median curves are given. For our purposes, we want the mean estradiol curve because the experimental measurements of Hcy are means, not medians. So, we use a standard method of estimating the mean from the median and the 5th and 95th percentiles [30]. This clinical data is graphed in Panel B of Fig 2b. We used Matlab’s *spline* interpolation function to create model curves for estradiol. We call the time-dependent estradiol curves *E*2(*t*), with estradiol in nM and time in days.

During human pregnancy, serum estradiol increases about 100-fold [11]. Tulchinsky et al. [11] regularly measured estradiol concentrations from 8 to 42 weeks of pregnancy. We performed cubic spline interpolation of the data [11] with Matlab’s *spline* function to obtain $\bar{E2}\left(t\right)$, our time-dependent estradiol curve during pregnancy, with estradiol in nM and time in days.

*E*2(*t*) and $\bar{E2}\left(t\right)$ are used as input functions for Eqs (2) and (3) to model time-dependent changes in PEMT and CBS enzyme activity levels due to estradiol variation. For example, in our model for pregnancy, $g(\bar{E2}\left(t\right))$ multiplies the *V*_*max*_ of PEMT, where $\bar{E2}\left(t\right)$ is the estradiol concentration on day *t* of pregnancy.

### Substrate inhibition

Most of the folate substrates in Fig 1 bind allosterically and reversibly to one or more enzymes in the folate cycle. For example, THF binds to DHFR, MS, SHMT, MTD, FTD and FTS and 5mTHF binds to DHFR, SHMT, MTD, and MTCH; see Table IV in [31]. When a folate substrate binds allosterically to a folate enzyme, the folate cannot be used as a substrate, and the enzyme cannot catalyze its reaction. Thus, substrate inhibition serves as a storage mechanism for both folates and folate enzymes [32]. If the total concentration of folate in the liver drops, many of these folate-enzyme complexes dissociate to produce more free folates and more free enzymes. In [31], we explained that this mechanism keeps most of the reaction velocities in the folate cycle near normal until total folate drops below 5*μ*M, where 20*μ*M is normal.

In “Folate deficiency” we discuss the effects of folate deficiency in the mathematical model with substrate inhibition. As in [31], we multiply the velocity of each reaction in the folate cycle by the factor

$$\begin{array}{c} \frac{A}{1+[THF]}, \end{array}$$ (5)

where *A* is chosen so that Eq (5) will be 1 at the male steady state value of THF. Details about Eq (5) can be found in [31].

We note that the steady state concentrations and velocities for males and females in this new mathematical model are similar to what we found using the 2018 model. For comparison, we have provided male and female values from both models in Fig B and Fig C of S1 Text. In addition, the complete MATLAB code is provided in S2 Text.

## Results and discussion

### Estrogen and homocysteine

In our previous paper [12], we examined how the differences in expression levels of SHMT, MTHFR, MS, BHMT, and PEMT in men and women affect the steady state levels of one-carbon metabolites. Here we investigate how one-carbon metabolites fluctuate during the menstrual cycle due to changes in estradiol. We begin with homocysteine (Hcy), a type of amino acid formed from methionine that has many deleterious effects. Its sulfur binds easily to proteins and can interfere with their functions [33]. In the plasma, Hcy is involved in blood clotting and in plaque formation [34] and is one of the major biomarkers for cardio-vascular disease [35, 36]. In addition, Hcy is involved in atherosclerosis [37] and is a biomarker for small for gestational age at birth (SGA), pre-eclampsia, and neural tube defects [38–41].

As shown in Panel B of Fig 2, there is a large variation in the estradiol curve in different women. The blue curve is the 95th percentile, the green curve is the mean and the red curve is the 5th percentile. The 5th and 95th percentile curves are taken from the BioCycle study [7] and the mean curve is estimated from these two percentiles and the median as indicated in Methods. These estradiol curves are very similar to the curves in [42, 43].

Estradiol affects Hcy in two major ways, by directly activating CBS and by upregulating PEMT, creating more betaine, which also activates CBS. The Hcy curves, computed by the model, corresponding to the three estradiol curves are shown in Panel A. As estradiol goes up, Hcy goes down and the changes are significant. These results correspond well with clinical observations. Tallova et al. [45] found that Hcy and estradiol vary inversely in plasma in menstruating women, a result also found by Michels et al. [44]. The Michels study had three time points, follicular, ovulatory, and luteal and the corresponding plasma Hcy concentrations are indicated as black dots in Panel A. The second two data points are right near our green mean Hcy curve. The first data point is below our green curve but the discrepancy is easy to understand. The mid-follicular phase in the BioCycle study [8] was day 7 to day 12 but the data points for this whole interval are combined at day 7. The women on days 9–11 would have been on the the sharply increasing portion of the estradiol curve. Thus the average estradiol in this group would be considerably higher than the estradiol in the model on day 7, and thus the data Hcy would have been lower than the value of the model Hcy curve at day 7. Interestingly, in a second study [46], Tallova et al. found that Hcy is higher in both the follicular phase and the luteal phase in depressed women than in controls, another indication of the importance of fluctuations of Hcy during the menstrual cycle.

### Homocysteine and oral contraceptives

Most of the estrogen in women during the child-bearing years is produced by developing follicles in the ovaries [47]. This naturally raises the question of homocysteine levels in women who take oral contraceptives (OCP), since those those pills, containing mostly progesterone and some estrogen, prevent follicular development by inhibiting GnRH production in the hypothalamus and FSH and LH production in the pituitary [47, 48]. The serum levels of estradiol of women taking the pill is 20–80 pg/ml which corresponds approximately to estradiol levels early in the follicular phase [49]. Using the model, we can compute the average estrogen over the menstrual cycle for normal women not taking the pill and women taking the pill and therefore can compute their average Hcy levels too; see Table 1.

**Table 1:** Homocysteine and the contraceptive pill in the mathematical model.

	Average E2	Average Hcy
menstruating women	0.44	1.29
women using OCPs	0.16	1.57
men	0.09	1.80

Thus the model shows that women using OCP will have higher Hcy on average than women not using OCP and this is borne out by the literature. Fallah et al [18] found an 80% increase in serum Hcy for women using OCPs and Momoni et al [19] found a 17% increase. Neither study controlled when the Hcy was measured for normal women, which as we have seen, makes a difference. Since Hcy is a major biomarker for cardiovascular disease, these results suggest that women who use OCPs may be at higher risk for cardiovascular events. Indeed, this is true. Kaminski et al. [50] found a 2-fold to 4-fold increased relative risk of arterial and venous thromboembolic events, and Carlton et al. [51] found that the relative risk for stroke increases by a factor of 2.75. The relative risk of cardiovascular events in women of age 20–50 years is quite low so these increases in risk are not too troubling. However, both studies point out that the increases in relative risks in the presence of other risk factors (such as smoking and high blood pressure) are much higher.

### Fluctuations in OCM during the menstrual cycle

**Fig 3:**
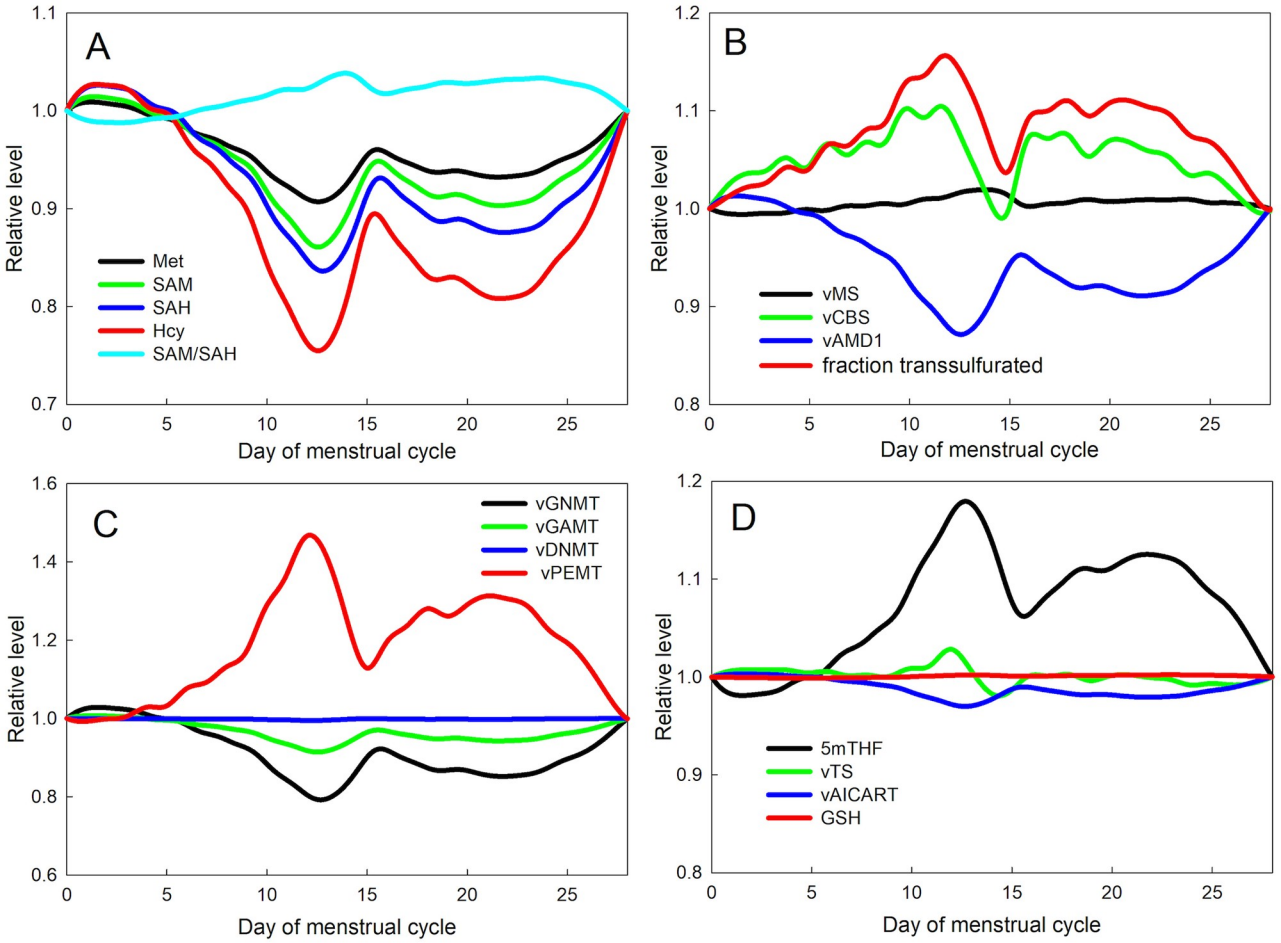
One-carbon metabolism during the menstrual cycle. Panel A shows the model concentrations of Met, SAM, SAH, and Hcy and the SAM/SAH ratio throughout the menstrual cycle, relative to their values at the beginning of the cycle. Panel B shows the model velocities of the MS, CBS, and AMD1 reactions, and the fraction of flux arriving from SAH that is transsulfurated. Panel C shows the model velocities of four important methyltransferases, GNMT, GAMT, DNMT, and PEMT. Finally, Panel D shows the relative model concentrations of 5mTHF and GSH, and the velocities of the TS and AICART reactions.

The four panels of Fig 3 show many of the metabolites and velocities in the folate and methionine cycles throughout the menstrual cycle as simulated in the model. Each curve is graphed relative to its value at the beginning of the menstrual cycle. The changes are caused by the changing values of estradiol (Fig 2). Panel A of Fig 3 shows the four methionine cycle metabolites, Met, SAM, SAH, and Hcy, that all move in concert in the opposite direction from the estradiol curve. It’s been observed experimentally that Met and E2 have an inverse relationship [10, 52]. And as remarked above, the changes in Hcy relative to E2 are confirmed by many clinical studies [44, 45]. The reasons for this behavior are clear: E2 stimulates CBS directly and also activates PEMT that raises the concentration of Bet, which also activates CBS. The activation of CBS draws mass out of the methionine cycle. The velocity of CBS follows the estradiol curve as shown in Panel B, which also shows that the velocity of the polyamine drain, vAMD1, follows the SAM curve as it should. We remark the the SAM/SAH ratio, often referred to as the “methylation capacity,” does not vary much during the cycle (cyan curve in Panel A).

Panel C shows the velocities of four important methyltransferases, GNMT, GAMT, DNMT, and PEMT. The most dramatic change is in PEMT since it is highly activated by E2, so, of course it follows the E2 curve. vGNMT and vGAMT drop as PEMT increases because they compete for molecules of SAM. On the other hand, the methylation of DNA, vDNMT, remains remarkably steady. This important homeostasis is achieved by the inhibition of MTHFR by SAM and the inhibition of GNMT by 5mTHF as we have explained elsewhere [53, 54]. Panel D shows that 5mTHF follows the shape of the E2 curve. The reason is that when E2 goes up, Hcy goes down, which lowers the velocity of the MS reaction causing 5mTHF to build up.

Perhaps, most interesting are the quantities that remain stable, or relatively stable, throughout the cycle. Although SAM and SAH change a lot, they change together, so the SAM/SAH ratio, known as the methylation capacity, doesn’t change much (Panel A). The velocity of the MS reaction doesn’t change much (Panel B) because as E2 rises, the corresponding drop in Hcy raises 5mTHF, which compensates for the drop in Hcy. As noted above, the long-range interactions stabilize the DNMT velocity, the DNA methylation rate (Panel C). Finally, Panel D shows that the concentration of glutathione, GSH, remains quite stable. There are two reasons. First, the concentration of GSH in liver cells is high, in the range 6000–7000*μ*M [55, 56], so fluctuations in the steady state velocity of CBS (approximately 31*μ*M/hr) that cause fluctuations in the amount of cysteine produced in the transsulfuration pathway will not change the relative amount of GSH very much. Secondly, GSH and the reduced version GSSG are exported rapidly to the blood where they are broken down rapidly to glycine, glutamate and cysteine and retransported back into the liver; details can be found in [24]. The net result is that the retransport of cysteine from the blood is much larger than the creation of cysteine by the transsufuration pathway from Hcy. Thus, fluctuations in the velocity of CBS do not affect GSH very much. Finally, we note that the velocity of the TS reaction (Panel D) follows the estradiol curve since TS is activated by estradiol (see Methods). We have not found evidence that AICART is activated by estradiol. It would make sense that these crucial reactions for making pyrimidines and purine would increase with estradiol because that would increase cell division in the uterus during the cycle.

Hcy sits at a crucial bifurcation point in the methionine cycle, since the flux arriving from SAH can be remethylated to reform methionine or transsulfurated by CBS, that is, sent down the transsulfuration pathway to make cysteine and glutathione. In Fig 1 it is shown that SAM activates CBS and inhibits BHMT. This is a mechanism for stabilizing total mass in the methionine cycle. Our simulations have demonstrated that as methionine input increases the fraction of flux arriving from SAH that is transsulfurated goes up, a result confirmed by [52] who measured the fraction transsulfurated in fasting and fed women. The red curve in Panel B shows that, even when methionine input is held constant, the fraction transsulfurated follows the estradiol curve and varies through the menstrual cycle.

### Is homocysteine a good biomarker for vitamin deficiencies?

**Fig 4:**
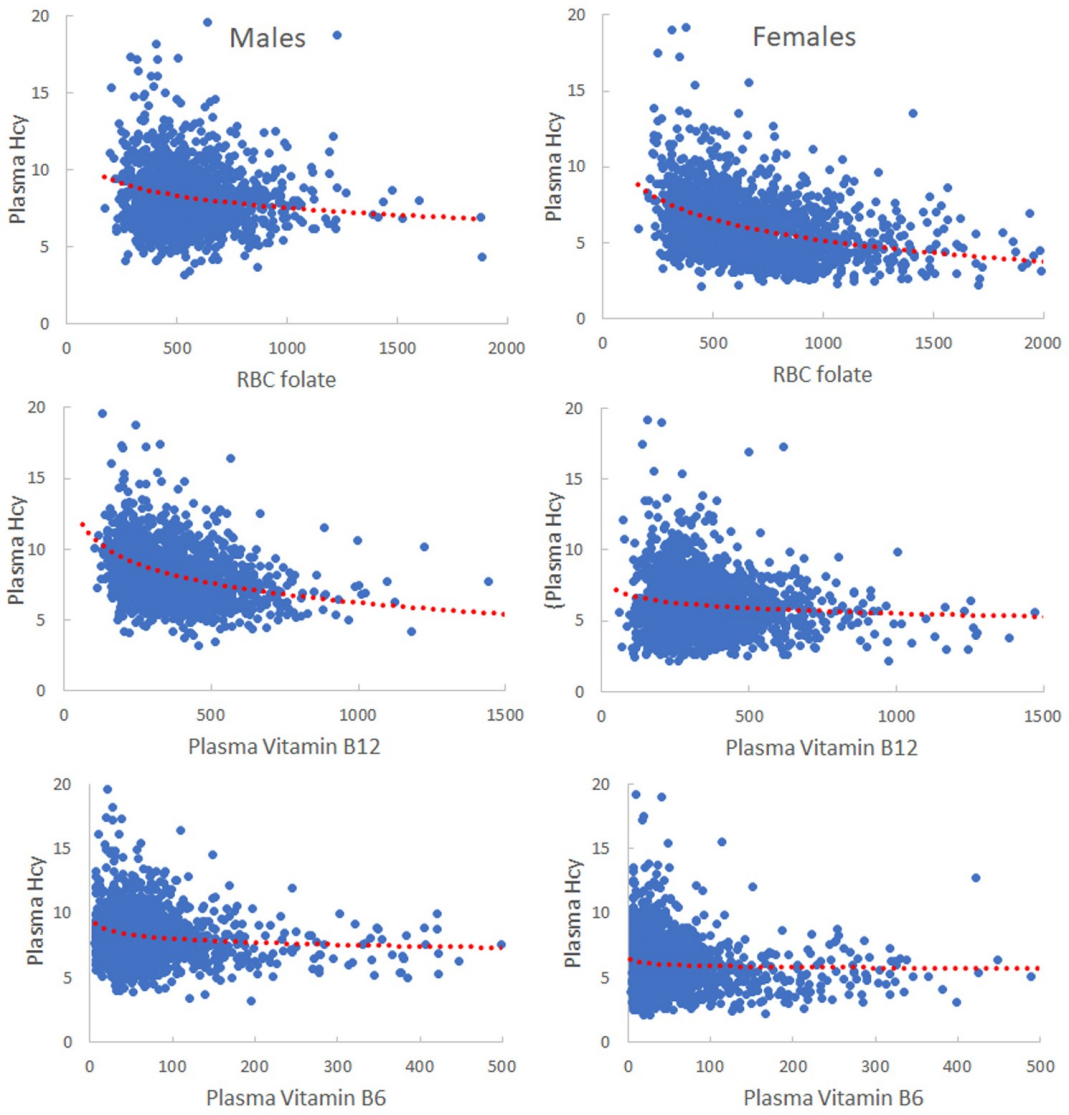
NHANES data on Hcy and vitamin deficiencies. These scatterplots lump together the NHANES studies for 2001–2006. Data is for men and women between ages 20 and 40. The red dotted curves show the mean Hcy concentration of vitamin bins of small size. RBC folate and *B*_6_ are in nanomolar, *B*_12_ is in picomolar, and plasma Hcy is in micromolar. RBC folate stands for red blood cell folate.

Homocysteine is created from SAH in the methionine cycle and then can be remethylated to form Met by BHMT and MS or sent down the transsulfuration pathway by CBS to make cysteine and glutathione (see Fig 1). It can also be converted to homocysteine-thiolactone [33]. Since Hcy has many deleterious effects, it is not surprising that there are many control mechanisms designed to keep its concentration low. These control mechanisms play an important role in determining the answer to the question posed in the title to this section. As we will see, Hcy does not vary very much in our model over wide ranges of vitamin concentrations and these results are supported by the National Health and Nutrition Examination Survey (NHANES) data in Fig 4 below. However, in our model, in the NHANES data, and in numerous research studies (discussed below), Hcy does increase rapidly for extremely low vitamin concentrations. Thus the answer to the motivating question is nuanced: Hcy is not a good indicator of vitamin status across a broad range of vitamin concentrations, but it is a biomarker for extremely low vitamin concentrations.

The pink boxes in Fig 1 are different forms of folate (vitamin *B*_9_) and 5mTHF is required for the MS reaction. Vitamin *B*_12_ is also required for the MS reaction. Vitamin *B*_6_ is required for two enzymes in in Fig 1, CBS and SHMT. Since the enzymes MS and CBS remove Hcy, it was natural for researchers to assume that deficiencies in the vitamins would lower the flux leading away from Hcy and thereby raise the concentration of Hcy. This mechanism is assumed by many of the authors in the fundamental Hcy reference edited by Carmel and Jacobsen [37]. However, one-carbon metabolism is very complicated and, as we shall see, concentrations are determined not only by local (in the diagram) changes but also system effects and the regulations indicated by the red arrows. As we will see, Hcy does not change very much as one varies the concentrations of folate, vitamin *B*_6_, and vitamin *B*_12_ over wide ranges, and we will explain why. In each case, the mechanisms are different.

#### Vitamin *B*_12_ deficiency

A plausible explanation of why *B*_12_ deficiency should raise Hcy concentration is that since the MS flux will be lower, the concentration of Hcy will be higher. This explanation is *false*. Most of the flux from Hcy to Met (via MS and BHMT) returns to Hcy after going around the methionine cycle and therefore doesn’t change the concentration of Hcy. We say “most of the flux” because there is a small loss of flux due to the polyamine pathway from SAM. If one removes the activating red arrow from SAM to CBS and the inhibiting red arrow from 5mTHF to GNMT, then as *B*_12_ concentration is lowered, the concentration of Hcy stays almost constant.

Lowering the amount of *B*_12_ does lower the MS flux dramatically. The question is what effect this will have on Hcy. Here there are two competing effects on Hcy. First, in *B*_12_ deficiency there is less MS flux and therefore less flux into SAM, so SAM concentration will decrease. When the SAM concentration decreases, that removes some of the activation of CBS so the Hcy concentration will tend to go up. Second, since there is less MS flux the concentration of 5mTHF will go up (the methyl trap) and that will increase the inhibition on GNMT. Less flux through GNMT will raise the concentration of SAM and that will increase the activation of CBS which will tend to lower the concentration of Hcy. Which one of these competing effects on Hcy dominates will depend on the details of the activation of CBS by SAM and the inhibition of GNMT by 5mTHF. But in some rough sense, the two effects cancel out which explains why Hcy concentration is relatively insensitive to changes in *B*_12_.

To see how these ideas play out in the model we multiply the velocity of the MS reaction by a constant *b*_12_ which ranges from 2 (twice normal) to 1 (normal), down to 0.1. Table 2 shows the effects of *B*_12_ deficiency in the model for males and females.

**Table 2:** The effects of *B*_12_ deficiency in males and females in the model.

Male						
*b*_12_	2	1	0.5	0.33	0.2	0.1
vMS	29.55	24.59	18.69	14.94	10.74	6.17
SAM	25.19	25.44	24.61	23.68	22.49	21.00
Hcy	1.82	1.80	1.86	1.93	2.06	2.17
5mTHF	2.60	4.66	7.72	9.82	12.17	14.70
vAICART	461.01	428.94	373.49	327.69	264.72	176.54
Female						
*b*_12_	2	1	0.5	0.33	0.2	0.1
vMS	36.94	29.62	21.89	17.32	12.36	7.11
SAM	21.33	22.46	22.46	21.88	20.87	19.54
Hcy	1.33	1.29	1.29	1.32	1.36	1.41
5mTHF	2.73	4.76	7.72	9.76	12.11	14.63
vAICART	508.64	474.93	416.24	366.64	296.40	197.85

As expected, *B*_12_ deficiency has a dramatic effect on *V*_MS_, the flux through the MS reaction. This creates a larger and larger methyl trap as folates collect in the form 5mTHF leaving fewer folates for the AICART reaction that makes purines. However the effect on Hcy concentration in the male is modest rising only from 1.82 when *b*_12_ = 1 to 2.17 when *b*_12_ = 0.1. In the female, the corresponding rise is even more modest (from 1.51 to 1.59) and this is what one sees in the NHANES data in Fig 4. The reason that the male Hcy is slightly more sensitive to *B*_12_ deficiency than the female is that males have less flux through GNMT normally (see S1 Text) so the inhibition by 5mTHF has less of an effect on SAM concentration.

The conclusion is that vitamin *B*_12_ deficiency is clinically significant because it creates a methyl trap which will dramatically lower the flux through the two crucial reactions, AICART and TS. However, you will not be able to detect low, medium, or high *B*_12_ by measuring Hcy, a fact born out by the NHANES data in Fig 4. Nevertheless, in the model (and in the NHANES data) extremely low B12 does cause Hcy to rise and therefore high Hcy could be caused by extremely low B12 and that possibility should be investigated.

#### Vitamin *B*_6_ deficiency

Vitamin *B*_6_ is a cofactor for the enzymes CBS and SHMT in the diagram in Fig 1. Since CBS removes Hcy and creates cystathionine, one would expect that *B*_6_ deficiency should raise Hcy concentration. However, this supposition is not borne out in the NHANES data in Fig 4 and, in fact, Gregory has remarked that “Fasting homocysteine concentration is not a good indicator of vitamin *B*_6_ status” [57]. Why is this? Again there are two competing effects. First, lowering vitamin *B*_6_ concentration should lower the CBS flux and raise Hcy. Secondly, we have shown [58] that lowering vitamin *B*_6_ concentration dramatically raises the serine concentration. Since serine is a co-substrate for the CBS reaction (see Fig 1) this will drive the CBS reaction faster and will tend to lower Hcy concentration. Again, the competing effects approximately cancel out, which explains the data in Fig 4 and Jesse Gregory’s remark.

The calculations in [58] were done with a much larger one-carbon model that included the folate cycle in the mitochondria and the entire transsulfuration pathway because *B*_6_ affects the mitochondrial enzymes SHMT and GDC (glycine decarboxylase), so the calculations are not repeated here with this smaller model that does not include the mitochondria. The increase in serine can be seen in Figure 1 of [58] where many confirming references from the literature are given.

#### Folate deficiency

**Fig 5:**
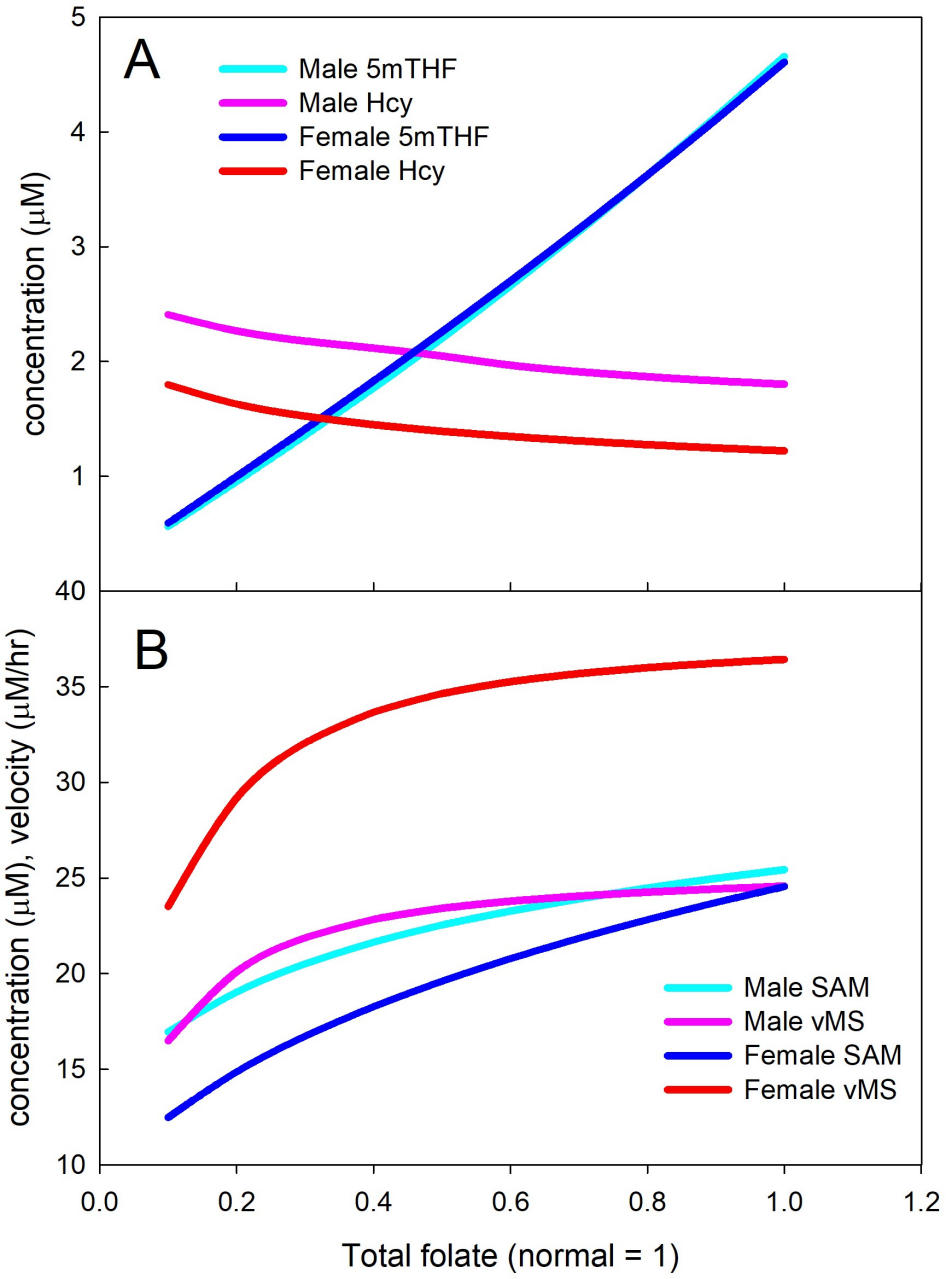
Effects of folate deficiency in the mathematical model. For both males and females, vMS, the velocity of the MS reaction, is homeostatic until total folate gets below 30% of normal. As a result, the Hcy concentration does not depend strongly on total folate. As discussed in [12], the *V*_*max*_ of the MS reaction is larger in females than in males, and as usual, the female Hcy concentration is below the male Hcy concentration. Both Hcy curves show the convex shape of the mean curves in the NHANES data in Fig 4a and 4b.

We explained in the Methods section that most of the folate substrates in Fig 1 bind allosterically and reversibly to one or more enzymes in the folate cycle. For example, THF binds to DHFR, MS, SHMT, MTD, FTD and FTS and 5mTHF binds to DHFR, SHMT, MTD, and MTCH. See Table IV in [31] and the references therein. When a substrate binds allosterically to an enzyme, it cannot be used as a substrate and the enzyme cannot be used to catalyze a reaction. This is a kind of group substrate inhibition [32] that is a storage mechanism both for folates and for folate enzymes. If the total concentration of folate in the liver drops, then many of these bound folate-enzyme complexes dissociate producing more free folates and more free enzyme. In [31], we explained that this mechanism keeps most of the reaction velocities in the folate cycle near normal until total folate drops below 5*μ*M, where 20*μ*M is normal. If we run our model without this substrate inhibition, then the Hcy concentration depends strongly on total folate; see Figure 5a in [12]. If we run our model with this group substrate inhibition we obtain the results in Fig 5. For both males and females, the velocity of the MS reaction is quite stable until total folate gets very low (Panel B). Even though 5mTHF declines linearly (Panel A) and total folate drops, the release of MS from the bound complexes compensates and the velocity stays stable. The concentration of Hcy changes only modestly over a wide range of folate deficiencies (Panel A), corresponding well to the NHANES data in Fig 4. Note that, as usual, the female Hcy concentration is below the male Hcy concentration. Both Hcy curves show the convex shape of the mean curve in the NHANES data in Fig 4a and 4b. Finally, the SAM concentration drops considerably because the drop in 5mTHF removes inhibition from GNMT so the flux through GNMT increases. The increased flux through GNMT raises SAH and increases Hcy (as pointed out by Davis et al. [59]). Much of the increase comes because higher GNMT flux lowers SAM thereby withdrawing activation of CBS.

We now return to the question of whether homocysteine is a good biomarker for vitamin deficiencies. It is worthwhile to start with clinical data. Fig 4 shows scatterplots from three NHANES studies showing the relationship between plasma Hcy and the three vitamins for both men and women. The red dotted curves show the Hcy means binned over small ranges of the vitamin concentrations. There clearly is an effect of vitamin concentration on Hcy but it is relatively small over a very wide range of vitamin concentrations. Notice that in all cases women have lower Hcy than men; we explained how this is caused by estrogen in [12]. The homeostasis of Hcy over wide ranges is caused by the mechanisms discussed above.

However, in all cases, both in the model and in Fig 4, Hcy rises rapidly for extremely low vitamin concentrations and this has been confirmed by numerous research studies. Yajnik et al. showed that *B*_12_ supplementation reduces hyper-homocysteinemia [60] and this was confirmed in [61]. Naurath et al. showed that supplementation by injections *B*_12_, *B*_6_, and folate lowered Hcy in the elderly by 10% [62]. And several authors have discussed the difficulties in diagnosis and the lack of agreed cutoffs for “deficiency” [63–65]. These difficulties are compounded by the very wide range of Hcy concentrations seen in populations for fixed *B*_12_, *B*_6_, and folate levels (see Fig 5). So, we believe that the answer to the question, “Is homocysteine a good biomarker for vitamin deficiencies,” is nuanced. Hcy concentration is not an indicator that concentrations of *B*_12_, *B*_6_, or folate are high, medium, or low. However, very high Hcy concentrations may indicate that *B*_12_, *B*_6_, or folate status is very low and therefore should be investigated further. Note that just because Hcy is not a good predictor for a wide normal range does not mean that vitamin differences in that range are not important. A *B*_12_ deficiency causes the methyl trap (accumulation of folates in the form 5mTHF) and greatly lowers the fluxes through the TS and AICART reactions that are necessary for cell division. *B*_6_ deficiency has serious consequences as outlined in [57, 58]. Folate deficiency decreases SAM and therefore will lower the flux through all the methylation reactions. So it is important to determine vitamin status over the wide normal range, one just can’t do it using Hcy.

### Homocysteine in pregnancy

As discussed above, E2 lowers Hcy through both direct and indirect activation of CBS. It is known that E2 increases dramatically during pregnancy by about 100-fold [11], and we would expect Hcy to decrease as a result. In fact, clinical data have shown that plasma Hcy concentrations drop by about 30% during pregnancy [52]. There are several explanations in the literature for why this is. Folic acid supplement use, hemodilution, and albumin decline are all known to contribute to decreased Hcy during pregnancy [38, 66]. It has been proposed that hormones may additionally have an important role in reducing Hcy during pregnancy [38, 66]. Elevated Hcy during early pregnancy is associated with a higher risk of pregnancy complications such as small for gestational age at birth (SGA), pre-eclampsia, and neural tube defects [38–41].

**Fig 6:**
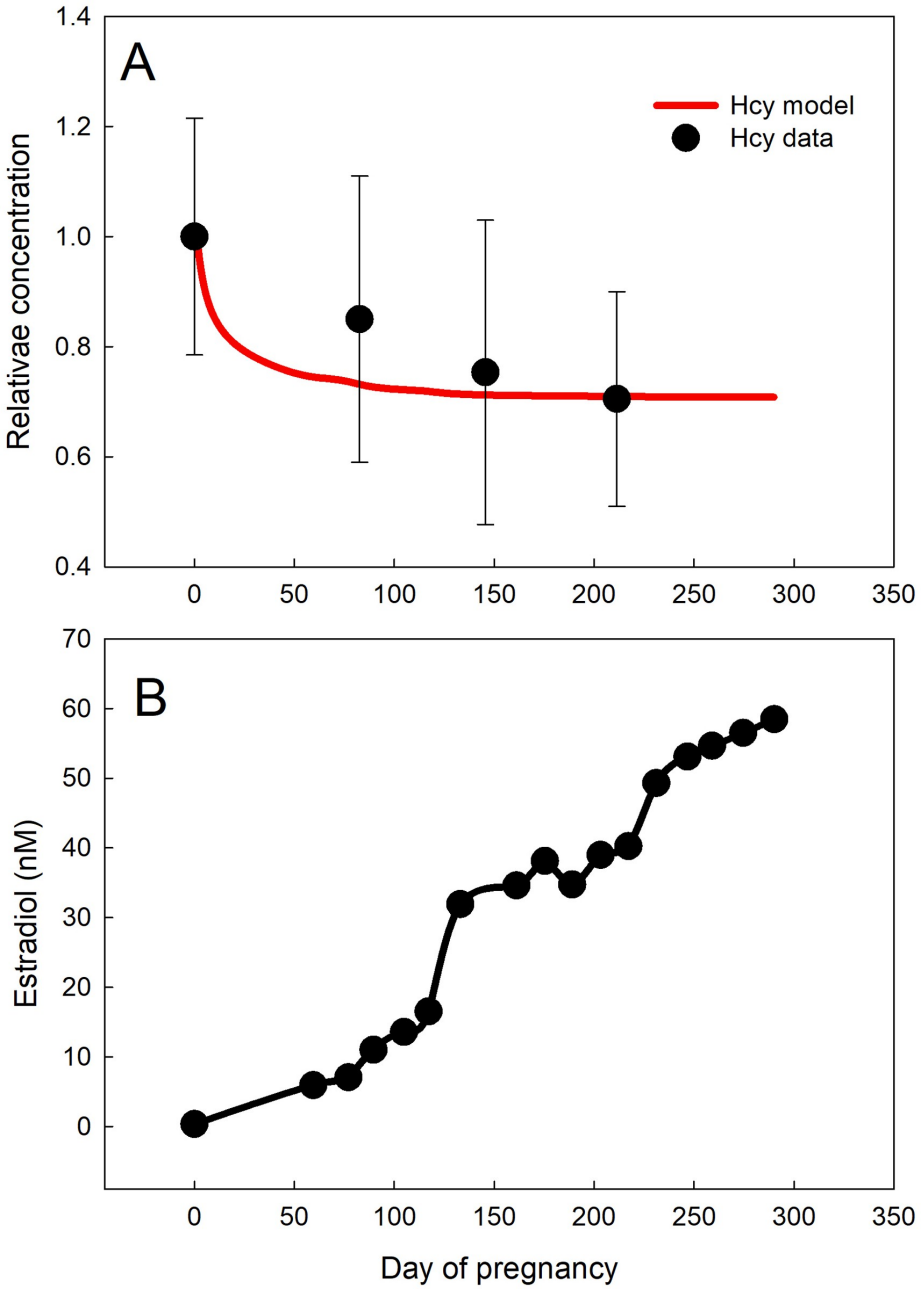
Homocysteine throughout pregnancy. Estradiol (E2) increases significantly from about 0.35 nM to 58.46 nM throughout pregnancy [7, 11]. The E2 data from [11] is reproduced in Panel B. Our model predicts Hcy throughout pregnancy as a result of E2 changes. Hcy is plotted relative to its concentration in menstruating women (red curve, Panel A). Our model corresponds well with data from [52], which is also plotted relative to Hcy in menstruating women (black dots, Panel A). The black bars indicate standard deviation.

We can use our mathematical model to better understand the role of E2 in lowering Hcy during pregnancy. Tulchinsky et al. [11] measured E2 in the plasma throughout human pregnancy, and this data is regraphed in Fig 6b. As described in the Methods, we used this data [11] to obtain an average E2 curve during pregnancy. In Fig 6a, we predict Hcy concentration over 280 days (40 weeks) of pregnancy and our results correspond well with experiments by Dasarathy et al. [52]. The Hcy data [52] was collected during each trimester of pregnancy, at approximately days 83, 146, and 211, and is plotted (black dots) with our model curve (red) in Fig 6a. The impact of E2 on Hcy during pregnancy is largest during the first trimester in both the data [52] and our model. The reason for this is that the effect of E2 on the activation of CBS saturates by the time E2 reaches 15–20 nM.

## Conclusion

We have enlarged and improved our mathematical model of sex differences in one-carbon metabolism so that we can study the time courses of of OCM metabolites during the menstrual cycle and pregnancy. The model predictions are consistent with experimental and clinical data and show the effects of the numerous homeostatic mechanisms in OCM. The most dramatic changes are in homocysteine. We have explained why menstruating women have lower homocysteine than men and we have shown how and why homocysteine varies during the menstrual cycle. We have also explained why homocysteine is not a good biomarker for vitamin *B*_12_, vitamin *B*_6_, and folate deficiencies and our conclusion is supported by NHANES data. Finally, we used the model to study homocysteine concentrations during pregnancy. Collection of clinical data on a finer time scale, for example days instead of just foliicular and luteal, would enable us to improve the model and make it even more useful. The complete mathematical model is described in S1 Text. In addition, the complete MATLAB code is in S2 Text so that other researchers can use it to run in silico experiments on sex differences in one-carbon metabolism.

## Supporting information

S1 Text Details of the full mathematical model. **Fig A**: Schematic diagram of the mathematical model. **Table A**: Variable names and usual acronyms. **Table B**: Constant concentrations and inputs in the model. **Table C**: Model kinetic parameters. **Fig B**: One-carbon metabolism for the male. **Fig C**: One-carbon metabolism for the female.
(PDF)

S2 Text MATLAB code for model simulations. (PDF)
