# Supplementary material for: One-carbon metabolism during the menstrual cycle and pregnancy
Source: PLoS Comput Biol. 2021 Dec 16;17(12):e1009708. doi: 10.1371/journal.pcbi.1009708 (PMC8741061; doi:10.1371/journal.pcbi.1009708)
Supplement: S1 Text — Fig A: Schematic diagram of the mathematical model. Table A: Variable names and usual acronyms. Table B: Constant concentrations and inputs in the model. Table C: Model kinetic parameters. Fig B: One-carbon metabolism for the male. Fig C: One-carbon metabolism for the female. (PDF) [file pcbi.1009708.s001.pdf]

Supplementary Methods

One-carbon metabolism during the menstrual cycle and pregnancy

R. Kim[1], H. F. Nijhout[2], M. Reed[1]

[1] Department of Mathematics, Duke University
[2] Department of Biology, Duke University

Corresponding author: Michael C. Reed, Department of Mathematics, Duke University, Durham, NC 27708. email: reed@math.duke.edu, phone: 919-660-2808, FAX: 919-660-2821.

In this supplementary material we provide details of the full mathematical model. Fig S1 shows a schematic diagram of the biochemical reactions in the model. Many of the equations in the model are the same as in our 2018 paper [1]. In this paper, we have added the direct influences of estradiol on PEMT, CBS, TS, and DHFR. All transmethylation pathways besides the GNMT, DNMT, GAMT, and PEMT reactions are modeled as "other." We have also added AMD1 and glutathione. Full substrate and enzyme names are given in the Figure A legend.

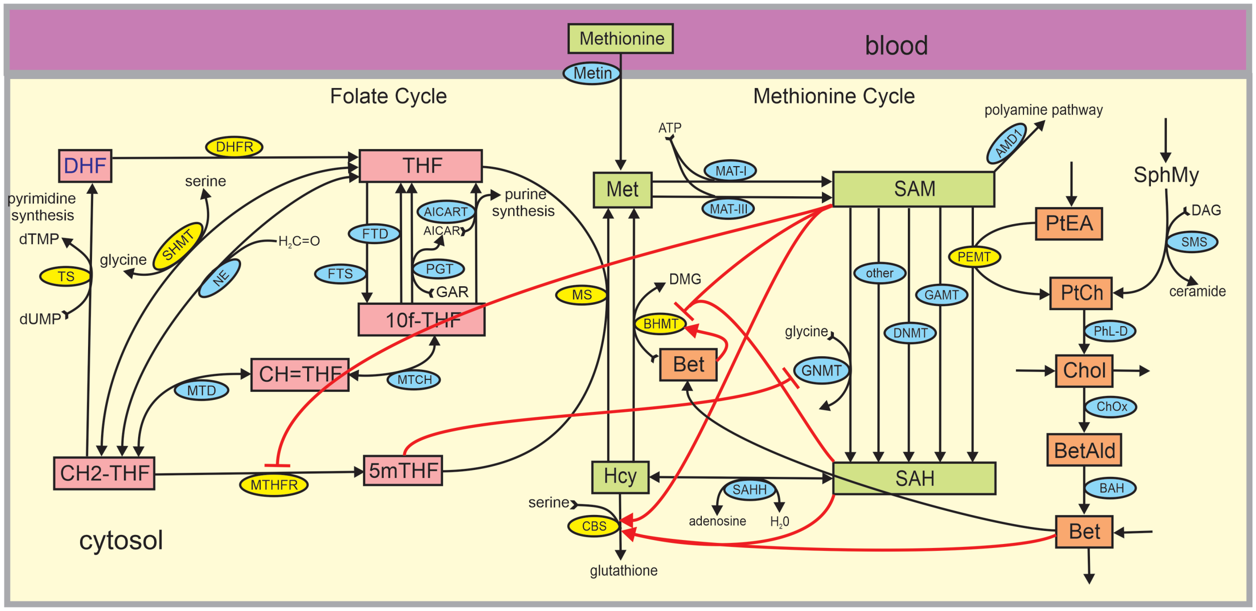

Figure A. One-carbon Metabolism. Substrates are indicated by rectangular boxes, green in the methionine cycle and red in the folate cycle. Each black arrow represents a biochemical reaction and the blue and yellow ellipses on the arrows contain the acronyms of the enzymes that catalyze the reactions. The yellow ellipses indicate the enzymes that are up- or down-regulated in females (see Table 1). Each red arrow is a long-range allosteric influence, either activation (arrow) or inhibition (bar). Substrate abbreviations: Met, methionine; SAM, S-adenosylmethionine; SAH, S-adenosylhomocysteine; Hcy, homocysteine; 5mTHF, 5-methyltetrahydrofolate; THF, tetrahydrofolate; 10fTHF, 10-formyltetrahydrofolate; DHF, dihydrofolate; CH2-THF, 5,10-methylenetrahydrofolate; CH=THF, 5,10-methenyltetrahydrofolate; SphMY, sphingomyelin; PtEA, phosphotidylethanolamine; Cho, choline; Bet-Ald, betaine aldehyde; Bet, betaine. Enzyme abbreviations: AICAR(T), aminoimidazolecarboxamide ribonucleotide (transferase); FTD, 10-formyltetrahydrofolate dehydrogenase; FTS, 10-formyltetrahydrofolate synthase; MTCH, 5,10-methylenetetrahydrofolate cyclohydrolase; MTD, 5,10-methylenetetrahydrofolate dehydrogenase; MTHFR, 5,10-methylenetetrahydrofolate reductase; TS, thymidylate synthase; SHMT, serine hydroxymethyltransferase; PGT, phosphoribosyl glycinamidetransformalase; DHFR, dihydrofolate reductase; NE, nonenzymatic interconversion of THF and 5,10-CH2-THF; MAT-I,methionine adenosyl transferase I; MAT-III, methionine adenosyl transferase III; AMD1, adenosylmethionine decarboxylase; GNMT, glycine N-methyltransferase; AS3MT, arsenic methyltransferase; PEMT, phosphotidylethanolamine methyltransferase; GAMT, gunadino-acetate methyltransferase; DNMT, DNA-methyltransferase; SAHH, S-adenosylhomocysteine hydrolase; CBS, cystathionine β-synthase; MS, methionine synthase; SMS, sphingomyelin synthase;

PhL-D, phospholipade D; ChOx, choline oxidase; BAH, betaine aldehyde dehydrogenase; BHMT, betaine-homocysteine methyltransferase.

In specifying the differential equations, we use lower case letters and simple abbreviations for the variables (substrates); these abbreviations are indicated in Table 1, below. Velocities are always indicated by V_{X} where the subscript X gives the acronym of the enzyme that catalyzes that particular velocity. Each velocity depends on the current values of substrates.

Table A, Variable names and usual acronyms

variable	usual acronym
met	MET
sam	SAM
sah	SAH
hcy	HCY
dhf	DHF
thf	THF
fthf	10f-THF
ch	CH=THF
ch2	CH2-THF
mthf	5mTHF
gnmt	GNMT
gnmtf	GNMT-5mTHF
fgnmtf	5mTHF-GNMT-5mTHF
bet	Bet
bet-ald	Bet-Ald
cho	Cho
pc	PtCho
gsh	GSH

The 18 differential equations are simply mass balance equations that say that the rate of change of the concentration of a substrate is the sum of the velocities of the reactions that make the substrate minus the sum of the reactions that use the substrate. Justifications for most of the model equations can be found in the 2018 paper [1], and all new additions are explained in the Methods. The full differential equations follow:

$$
\begin{aligned}
\frac{d}{dt}(met) &= metin(t) + V_{\mathrm{BHMT}}(hcy, bet, sam, sah) + V_{\mathrm{MS}}(mthf, hcy) - V_{\mathrm{MATI}}(met, sam) \\
&\quad - V_{\mathrm{MATIII}}(met, sam) \\
\frac{d}{dt}(sam) &= V_{\mathrm{MATI}}(met, sam) + V_{\mathrm{MATIII}}(met, sam) - V_{\mathrm{GNMT}}(sam, sah, gly, gnmt, gnmtf) \\
&\quad - V_{\mathrm{PEMT}}(sam, sah, pe) - V_{\mathrm{GAMT}}(sam, sah, gaa) - V_{\mathrm{DNMT}}(sam) - V_{\mathrm{other}}(sam, sah) \\
&\quad - V_{\mathrm{AMD1}}(sam) \\
\frac{d}{dt}(sah) &= V_{\mathrm{GNMT}}(sam, sah, gly, gnmt, gnmtf) + V_{\mathrm{other}}(sam, sah) + V_{\mathrm{PEMT}}(sam, sah, pe) \\
&\quad + V_{\mathrm{GAMT}}(sam, sah, gaa) + V_{\mathrm{DNMT}}(sam) - V_{\mathrm{SAAH}}(sah, hcy) \\
\frac{d}{dt}(hcy) &= V_{\mathrm{SAAH}}(sah, hcy) - V_{\mathrm{BHMT}}(hcy, bet, sam, sah) - V_{\mathrm{MS}}(mthf, hcy) \\
&\quad - V_{\mathrm{CBS}}(hcy, bet, sam, sah, ser) \\
\frac{d}{dt}(dhf) &= V_{\mathrm{TS}}(dump, ch2) - V_{\mathrm{DHFR}}(dhf, nadph) \\
\frac{d}{dt}(thf) &= V_{\mathrm{DHFR}}(dhf, nadph) + V_{\mathrm{MS}}(mthf, hcy) + V_{\mathrm{FTD}}(fthf) + V_{\mathrm{PGT}}(fthf, gar) \\
&\quad + V_{\mathrm{AICART}}(fthf, aic) - V_{\mathrm{FTS}}(thf, hcooh, fthf) - V_{\mathrm{SHMT}}(ser, thf, gly, ch2) \\
&\quad - V_{\mathrm{NE}}(thf, hcoh, ch2) \\
\frac{d}{dt}(fthf) &= V_{\mathrm{MTCH}}(ch, fthf) + V_{\mathrm{FTS}}(thf, hcooh, fthf) - V_{\mathrm{PGT}}(fthf, gar) \\
&\quad - V_{\mathrm{AICART}}(fthf, aic) - V_{\mathrm{FTD}}(fthf) \\
\frac{d}{dt}(ch) &= V_{\mathrm{MTD}}(ch2, ch) - V_{\mathrm{MTCH}}(ch, fthf) \\
\frac{d}{dt}(ch2) &= V_{\mathrm{SHMT}}(ser, thf, gly, ch2) + V_{\mathrm{NE}}(thf, hcoh, ch2) - V_{\mathrm{TS}}(dump, ch2) \\
&\quad - V_{\mathrm{MTD}}(ch2, ch) - V_{\mathrm{MTHFR}}(ch2, nadph, sam, sah) \\
\frac{d}{dt}(mthf) &= V_{\mathrm{MTHFR}}(ch2, nadph, sam, sah) - V_{\mathrm{MS}}(mthf, hcy) + k_2(gnmtf) \\
&\quad - 2k_1(mthf)(gnmt) + k_4(fgnmtf) - k_3(mthf)(gnmtf) \\
\frac{d}{dt}(gnmt) &= k_2(gnmtf) - 2k_1(mthf)(gnmt) \\
\frac{d}{dt}(gnmtf) &= -k_2(gnmtf) + 2k_1(mthf)(gnmt) - k_3(mthf)(gnmtf) + k_4(fgnmtf) \\
\frac{d}{dt}(fgnmtf) &= k_3(mthf)(gnmtf) - k_4(fgnmtf)
\end{aligned}
$$

(continued on next page)

$$\frac{d}{dt}(bet) = betin + V_{\mathrm{BAH}}(bet\text{–}ald) - V_{\mathrm{BHMT}}(hcy, bet, sam, sah) - (.0096)(bet)$$

$$\frac{d}{dt}(bet\text{–}ald) = V_{\mathrm{CHO}}(cho) - V_{\mathrm{BAH}}(bet\text{–}ald) - (.01)(bet\text{–}ald)$$

$$\frac{d}{dt}(cho) = cholin + V_{\mathrm{PPL}}(pc) - V_{\mathrm{CHO}}(cho) - cholout(cho)$$

$$\frac{d}{dt}(pc) = V_{\mathrm{PEMT}}(sam, sah, PtEA) + V_{\mathrm{SMS}}(Sphmy) - V_{\mathrm{PPL}}(pc)$$

$$\frac{d}{dt}(gsh) = V_{\mathrm{CBS}}(hcy, bet, sam, sah, ser) - (0.015)(gsh)$$

Some of the reactions depend on the concentrations of other substrates that are not variable (in the model) and are assumed to be constant. These are give in Table B.

Table B. Constant concentrations (μM) and inputs (μM/hr) in the model

abbreviation	value	name
aic	2.1	AICARP
dump	20	DUMP
gly	1850	glycine
gaa	10	guanadinoacetate
gar	10	GAR
hcoh	500	HCOH (formaldehyde)
hcooh	900	HCOOH (formate)
ias	1	inorganic arsenic
nadph	50	NADPH
PE	10	phosphotidylethanolamine
ser	468	serine
SphMy	10 (13 in females)	sphingmyelin
metin	40	methionine input
cholin	200	choline input
betin	13	betaine input

The details of the biochemistry and the biology are in the functional forms that show how each of the velocities depends on the current values of the variables that influence it. Many reactions have Michaelis-Menten kinetics in one of the following standard forms:

$$V = \frac{V_{max}[S]}{K_m + [S]}, \qquad V = \frac{V_{max}[S_1][S_2]}{(K_{S_1} + [S_1])(K_{S_2} + [S_2])}$$

$$V = \frac{V^f_{max}[S_1][S_2]}{(K_{S_1} + [S_1])(K_{S_2} + [S_2])} - \frac{V^b_{max}[P_1][P_2]}{(K_{P_1} + [P_1])(K_{P_2} + [P_2])}$$

for unidirectional, one substrate, unidirectional, two substrates, and bidirectional, two substrates, two products, respectively. For these reactions, Table C lists the K_m and V_{max} values. In general, we take K_m values from the literature. V_{max} values are extremely variable because they depend on enzyme expressions levels that vary in time and therefore experimental measurements *in vivo* are difficult and unreliable. We usually adjust the V_{max} values so as to obtain the typical substrate concentration values that we find in the literature. Parameters have sometimes been chosen by comparing model outputs in various circumstances to qualitative and quantitative experimental data.

Table C. Model kinetic parameters (time in hrs, concentration in μM)

parameter	literature	model	reference
**AICART**			
$K_{m,fthf}$	5.9-50	5.9	[2][3][4][5]
$K_{m,aicar}$	10-100	100	[2][3][5]
V_{max}		45000	
**AMD1**			
K_m	210-286	245	[6]
V_{max}		100	
**BAH**			
K_m	214-306	250	[7]
V_{max}		45	
**CHOx**			
K_m	140-270	200	[8]
V_{max}		125	
**DHFR**			
$K_{m,dhf}$	0.12-1.9	0.5	[3][5][9][10]
$K_{m,nadph}$	0.3-5.6	4.0	[3][5][9][10]
V_{max}	350-23000	5000	[3][5][9]
**FTD**			
$K_{m,fthf}$	0.9-20	20	[11][12]
V_{max}		14,000	
**FTS**(forward direction from thf to fthf)			
$K_{m,thf}$	0.1-600	3	[4][5]
$K_{m,hcooh}$	8-1000	43	[4][5]
V_{max}	100-468000	2000	[4][5]
**MS**			
$K_{m,mthf}$	22-34	25	[13][14]
$K_{m,hcy}$	0.1-6	1	[15]
V_{max}		244	[15]
**MTCH**(forward direction from ch to fthf)			
$K_{m,ch}$	4-250	250	[3][4][5]

V_{max}	880-1380000	800000	[3][4]
$K_{m,fthf}$	20-450	100	[3][4][5]
V_{max}	10.5-1380000	20000	[3][4]
**MTD**(positive direction from ch2 to ch)			
$K_{m,ch2}$	2-5	2	[4][5]
V_{max}	520-594000	200000	[9][4][5]
$K_{m,ch}$	1-10	10	[4][16]
V_{max}	594000	594000	[4]
**PGT**			
$K_{m,fthf}$	4.9-58	4.9	[5][3][17][18]
$K_{m,gar}$	520	520	[5][3][17][18]
V_{max}	6600-16200	16200	[5][3][17][18]
**PhL-D**			
$K_{m,sphmy}$	18-20.3	400	[19]
V_{max}		525	
**SAHH**(forward direction from SAH to Hcy)			
$K_{m,sah}$	0.75-15.2	6.5	[20][21][22][23]
V_{max}		448	
$K_{m,hcy}$	56.6-200	150	[21][22][24]
V_{max}		755	
**SHMT**(positive direction is from thf to ch2)			
$K_{m,ser}$	350-1300	600	[3][4][5][25]
$K_{m,thf}$	45-300	50	[3][4][5][26]
V_{max}	500-162000	40000	[3][4][26]
$K_{m,gly}$	3000-10000	3000	[3][4][5][25][9]
$K_{m,ch2}$	3000-10000	3200	[3][4][9][26]
V_{max}	12600-120000000	2500000	[3][4][9]
**SMS**			
$K_{m,sphmy}$	18-20.3	20	[27][28]
V_{max}		30	

The effects of estradiol on V_{PEMT}, V_{CBS}, V_{TS}, and V_{DHFR} and the new form of V_{CBS} are discussed in detail in the Methods. All other reactions with nonstandard kinetics follow, and have been justified in [1].

BHMT. The velocity of the BHMT reaction is given by

$$V_{\mathrm{BHMT}}((hcy),(bet),(sam),(sah)) = \left(\frac{V_{max}(hcy)(bet)}{(K_m^{hcy}+(hcy))(K_m^{bet}+(bet))}\right) \cdot \left(\frac{e^{-.0021((sam)+(sah))}}{e^{-.0021(32.3)}}\right) \cdot \left(1+\frac{(0.16)((bet)-315)}{315}\right).$$

parameter	value	reference
V_{max}	1600	
K_m^{hcy}	12	[29]
K_m^{bet}	2000	[30]

DNMT. The velocity of the DNMT reaction is given by

$$V_{\mathrm{DNMT}}(sam, sah) = \frac{V_{max}(sam)}{K_m(1+\frac{(sah)}{K_i})+(sam)}.$$

parameter	value	reference
V_{max}	5	
K_m	1.4	[31]
K_i	1.4	[31]

GAMT. The velocity of the GAMT reaction is given by

$$V_{\mathrm{GAMT}}(sam, sah, gaa) = \frac{V_{max}(sam)(gaa)}{(K_m(1+\frac{sah}{K_i})+sam)(K_m+gaa)}.$$

where $V_{max} = 144$ for males and $V_{max} = 117$ for females because a higher percentage of body mass is muscle in males.

parameter	value	reference
K_m	49	[32]
K_i	16	[32]

GNMT. The velocity of the GNMT reaction is given by

$$V_{\text{GNMT}}(sam, sah, gnmt, gnmtf) \;=\; \frac{V_{max}(sam)}{(K_m(1+\frac{sah}{K_i})) + (sam)])}.$$

where $V_{max} = (525)(gnmt + (.5)(gnmtf))$ for males and $V_{max} = (1225)(gnmt + (.5)(gnmtf))$ for females. gnmt represents the concentration of free enzyme and gnmtf represents the concentration of enzyme with one molecule of 5mTHF bound to it. These details are not indicted in Figure 1 but can be found, with justifications, in [33].

parameter	value	reference
K_m	100	[32]
K_i	35	[32]

MAT-I. The velocity of the MAT-I reaction is given by

$$V_{\text{MAT-I}} \;=\; \left(\frac{V_{max}(met)}{K_m + met)}\right)(0.23 + (0.8)e^{-(0.0026)(sam)}).$$

parameter	value	reference
V_{max}	260	[34]
K_m	41	[34]

MAT-III. The velocity of the MAT-III reaction is given by

$$V_{\text{MAT-III}} \;=\; \left(\frac{V_{max}(met)^{1.21}}{K_m + (met)^{1.21}}\right)\left(1 + \frac{(7.2)(sam)^2}{K_a^2 + (sam)^2}\right).$$

parameter	value	reference
V_{max}	220	[35]
K_m	300	[35]
K_a	360	[34]

MTHFR. The velocity of the MTHFR reaction is given by

$$V_{\text{MTHFR}}(ch2, nadph, sam, sah) \;=\; \left(\frac{V_{max}(ch2)(nadph)}{(K_{m,1} + ch2)(K_{m,2} + nadph)}\right)\left(3 \cdot \frac{10}{10 + (sam - sah)}\right).$$

parameter	value	reference
V_{max}	2000	[36, 37, 38]
$K_{m,1}$	50	[36, 37, 38]
$K_{m,2}$	16	[36, 37, 38]

NE. The kinetics of the non-enzymatic reversible reaction between thf and ch2 are taken to be mass action,

$$V_{\mathrm{NE}} \;=\; k_1(thf)(hcho) - k_2(ch2),$$

with rate constants are $k_1 = 0.15$, and $k_2 = 12$. $hcho$ represents formaldehyde, which is a constant in the program.

Other transmethylation fluxes. We treat all other fluxes in the transmethylation pathway as “other” and give this pathway “average” kinetic parameters.

$$V_{\mathrm{other}}(sam, sah) \;=\; \frac{V_{max}(sam)}{K_{m,1}(1+\frac{(sah)}{K_{m,2}}) + (sam)}$$

where $V_{max} = 56$, $K_{m,1} = 50$, and $K_{m,2} = 15$.

PEMT. The velocity of the PEMT reaction is given by

$$V_{\mathrm{PEMT}}(sam, sam, pe) \;=\; \frac{V_{max}(sam)}{(K_{m,1} + (sam))(1+\frac{sah}{K_i})}\frac{pe}{(K_{m,2}+pe)}.$$

parameter	value	reference
V_{max}	1470	
$K_{m,1}$	18.2	[32]
K_i	3.8	[32]
$K_{m,2}$	5000	[39]

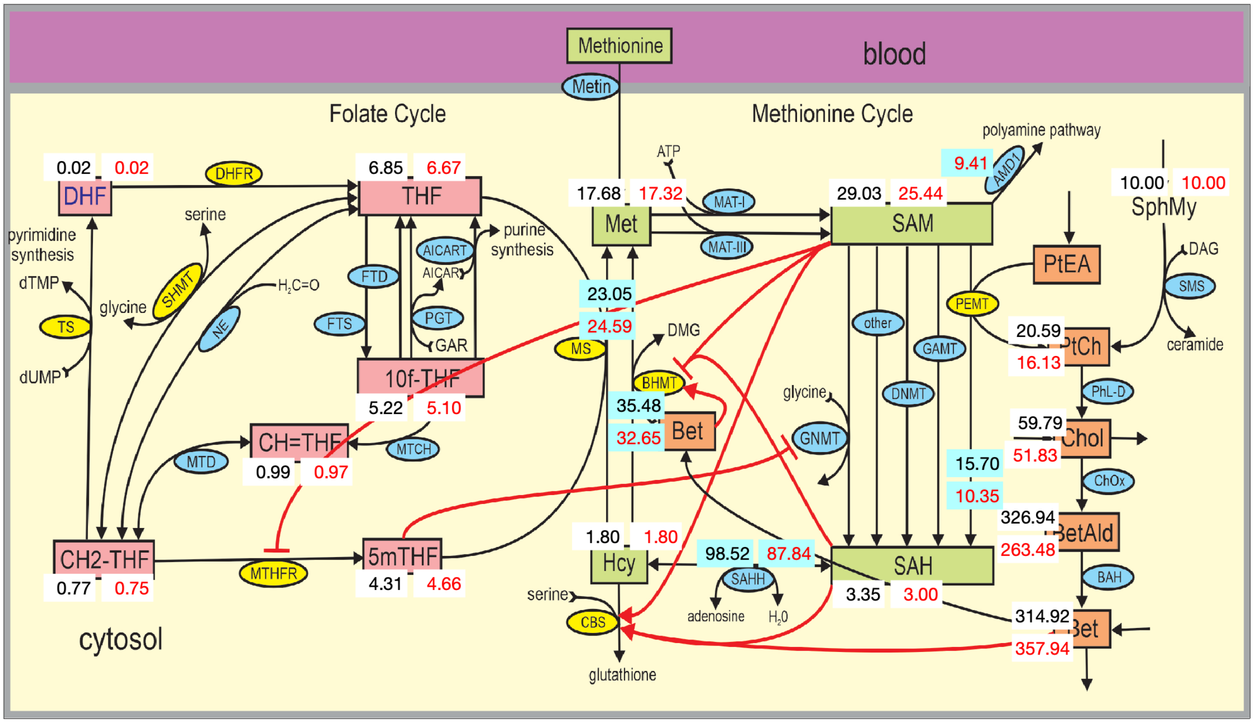

Figure B. One-carbon metabolism for the male. The black numbers indicate male model values from the 2018 paper [1], and the red numbers indicate male model values from this paper. The numbers in the white boxes are concentrations in micromolar. The numbers in the blue boxes are fluxes in micromolar/hour.

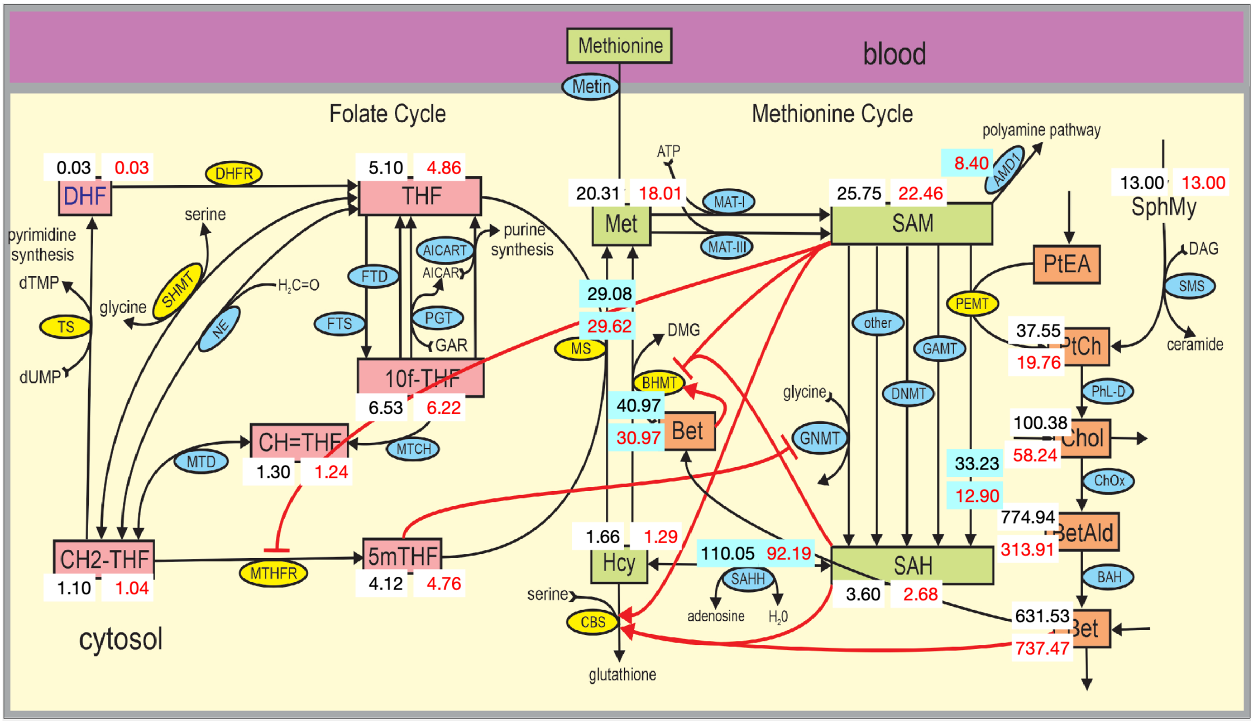

Figure C. One-carbon metabolism for the female. The black numbers indicate female model values from the 2018 paper [1], and the red numbers indicate female model values from this paper.

The numbers in the white boxes are concentrations in micromolar. The numbers in the blue boxes are fluxes in micromolar/hour.

References

[1] Sadre-Marandi F, Dahdoul T, Reed MC, Nijhout HF: **Sex differences in hepatic one-carbon metabolism**. *BMC Systems Biology* 2018.

[2] Rayl EA, Moroson BA, Beardsley GP: **The human purH gene product, 5-aminoimodazole-4-carboxamide ribonucleotide formyltransferase/IMP cyclohydrolase. Cloning, sequence, expression, purification, kinetic analysis, and domain mapping**. *J. Biol. Chem.* 1996, **271**:2225–2233.

[3] Seither R, Trent DF, Mickulecky DC, Rape TJ, Goldman ID: **Folate-pool interconversions and inhibition of biosynthetic processes after exposure of L1210 leukemia cells to antifolates**. *J. Biol. Chem.* 1989, **264**:17016–17023.

[4] Strong WB, Tendler SJ, Seither RL, Goldman ID: **Purification and Properties of Serine Hydroxymethyltransferase C1-Tetrahydrofolate Synthase from L1210 Cells**. *J. Biol. Chem.* 1990, **265**:12149–12155.

[5] Vorontzov IN, Greshilov MM, Belousova AK, Gerasimova GK: **Mathematical description and investigation of the principles of functioning of the folic acid cycle**. *Biokhimiya* 1980, **45**:83–97.

[6] White EL, Arnett G, III JAS, Shannon WM: **Characterization of S-adenosylmethionine decarboxylase induced by human cytomegalovirus infection**. *Virus Research* 1994, **31**:255–263.

[7] Chern M, Gage D, Pietruszko R: **Betaine aldehyde, betaine, and choline levels in rat livers during ethanol metabolism**. *Bioche. Pharmacol.* 2000, **60**:1629–1637.

[8] Zhang J, Blusztajn J, Zeisel S: **Measurement of the formation of betaine aldehyde and betaine in rat liver mitochondria by high pressure liquid chromatography-radioenzymatic assay**. *Biochem. Biophys. Acta* 1992, **1117**:333–339.

[9] Jackson RC, Harrup KR: **Studies with a mathematical model of folate metabolism.** *Arch. Biochem. Biophys.* 1973, **158**:827–841.

[10] Blake RL: **Eukaryotic dihydrofolate reductase**. *Adv. Enzymol.* 1995, **60**:23–.

[11] Schirch D, Villar E, Mara B, Barra D, Schrich V: **Domain structure and function of 10-formyltetrahydrofolate dehydrogenase**. *J. Biol. Chem.* 1994, **269**:24728–24735.

[12] Kim DW, Huang T, Schirch D, Schrich V: **Properties of Tetrahydropteroylpentaglutamate bound to 10-formyltetrahydrofolate dehydrogenase**. *Biochem.* 1996, **35**:15772–15783.

[13] Finkelstein JD, Martin JJ: **Methionine metabolism is mammals: Adaptation to methionine excess**. *J. Biol. Chem.* 1986, **261**:1582–1587.

[14] Banerjee R, Frasca V, Ballou D, Matthews R: **Participation of Cob(I)alamin in the reaction catalyzed by methionine synthase from Escherichia coli: a steady state and rapid reaction kinetic analysis,**. *Biochem.* 1990, **29**:11101–11109.

[15] Banerjee R, Chen Z, Gulati S: **Methionine synthase from pig liver**. *Mewth. Enzymol.* 1997, **281**:189–197.

[16] Wagner C: *Folate in Health and Disease*, New York: Marcel Dekker 1995 chap. Biochemical role of folate in cellular metabolism, :23–42.

[17] Caperelli CA: **Mammalian glycinamide ribonucleotide transformylase: purification and some properties**. *Biochemistry* 1985, **24**:1316–1320.

[18] Caperelli CA: **Mammalian glycinamide ribonucleotide transformylase. Kinetic mechanism and associated de novo purine biosynthetic activities**. *J. Biol. Chem.* 1989, **264**:5053–5057.

[19] Vinggaard A, Hunsen H: **Characterization and partial purification of phospholipase D from human placenta**. *Biochim Biophys Acta* 1995, **1258**:169–176.

[20] Doskeland SO, Ueland PM: **Comparison of some physicochemical and kinetic properties of S-adenosylhomocysteine hydrolase from bovine liver, bovine adrenal cortex and mouse liver**. *Biochim Biophys Acta* 1982, **708**:185–193.

[21] Fujioka M, Takata Y: **S-Adenosylhomocysteine Hydrolase from rat liver: Purification and some properties**. *J. Biol. Chem.* 1981, **256**:1631–1635.

[22] Hershfield M, Aiyar VN, Premakumar R, Small WC: **S-Adenosylhomocysteine hydrolase from human placenta**. *J. Biochem.* 1985, **230**:43–52.

[23] Klor D, Kurz J, Fuch S, Faust B, Osswald H: **S-adenostlhomocysteine-hydrolase from bovine kidney: enzymatic and binding properties**. *Kid. Blood Press. Res.* 1996, **19**:100–108.

[24] Gomi T, Takata Y, Date T, Motoji F, Akasamit RR, Backlund P, Cantoni G: **Site-directed mutagenesis of rat liver S-Adenosylhomocysteine**. *J. Biol. Chem.* 1990, **265**:16101–16107.

[25] Schirch V, Hopkins S, Villar E, Angelaccio S: **Serine hydroxymethyltransferase from Escherichia coli: purification and properties**. *J. Bacteriol.* 1985, **163**:1–7.

[26] Schirch V: **Purification and properties of folate-dependent enzymes from rabbit liver**. *Meth. Enzymol.* 1997, **281**:146–161.

[27] Krut O, Wigmann K, Kashkar A, Yazdanpanah B, Kronke M: **Novel tumor necrosis factor-responsive mammalian neutral sphygomylenase-3 is a C-tail-anchored protein**. *J. Biol. Chem.* 2006, **281**:13784–13793.

[28] Kim S, Ahn K, Jeon H, Lee D, Jung S, Jung K, Kim D: **Purification of neutral sphingomyelinase 2 from bovine brain and its calcium-dependent activation**. *J. Neurochemistry* 2010, **112**:1088–1097.

[29] Finkelstein JD, Harris BJ, Kyle WE: **Methionine metabolism in mam- mals: kinetic study of betaine-homocysteine methyltransferase**. *Arch. Biochem. Biophys.* 1972, **153**:320–324.

[30] Jiracek J, Collinsova M, Rosenberg I, Budesinsky M, Protivinska E, Netusilova H, Garrow T: **S-alkylated homocysteine derivatives: New inhibitors of human betaine-homocysteine s-methyltransferase**. *J. Med. Chem.* 2006, **49**:3982–3989.

[31] Flynn J, Reich N: **Murine DNA (cytosine-5-)-methyltransferase: Steady-state and substrate trapping analyses of the kinetic mechanism**. *Biochemistry* 1998, **37**:15162–15169.

[32] Clarke S, Banfield K: *Homocysteine in Health and Disease (Ed. R. Carmel and D. W. Jacobsen)*, Cambridge University Press. 7 2001 chap. S-Adenosylmethionine-dependent methyltransferases.

[33] Reed M, Gamble M, Hall M, Nijhout H: **Mathematical analysis of the regulation of competing methyltransferases.** *BMC Systems Biology* 2015, **9**:69–.

[34] Sullivan DM, Hoffman JL: **Fractionation and kinetic properties of rat liver and kidney methionine adenosyltransferase isozymes**. *Biochem.* 1983, **22**:1636–1641.

[35] SanchezdelPino MM, Corrales FJ, Mato JM: **Hysteretic Behavior of Methionine Adenosyltransferase III: methionine switches between two conformations of the enzyme with different specific activity**. *J. Biol. Chem.* 2000, **275**:23476–23482.

[36] Matthews RG: **methylenetetrahydrofolate reductase from pig liver**. *Meth. Enzymol.* 1986, **122**:372–381.

[37] Green JM, MacKensie RE, Matthews RG: **Substrate flux through methylenetetrahydrofolate dehydrogenase: Predicted effects of the concentration of methylenetetrahydrofolate on its partitioning into pathways leading to nucleotide biosynthesis or methionine regeneration**. *Biochem.* 1988, **27**:8014–8022.

[38] Daubner SC, Matthews RG: **Purification and properties of methylenetetrahydrofolate reductase from pig liver**. *J. Biol. Chem.* 1982, **257**:140–145.

[39] Vance DE, Ridgway ND: **The methylation of phosphatidylethanolamine**. *Prog. Lip. Res.* 1988, **27**:61–79.
