# Supplementary material for: One-carbon metabolism during the menstrual cycle and pregnancy
Source: PLoS Comput Biol. 2021 Dec 16;17(12):e1009708. doi: 10.1371/journal.pcbi.1009708 (PMC8741061; doi:10.1371/journal.pcbi.1009708)
Supplement: S2 Text — (PDF) [file pcbi.1009708.s002.pdf]

```
% One-carbon metabolism during the menstrual cycle and pregnancy
% Kim, Nijhout, Reed (2021)
% MATLAB code

%% steady state simulations
p = parameters;

%%% set female params %%%
% (comment out for male)
p.val(p.pf) = 1;
p.val(p.e2) = 0.4368; % mean e2
p.val(p.gnmtv) = 0.7; %male=0.3
p.val(p.gamtv) = 1.3; %male=1.6
%%%%%%%%%%%%%%%%%%%%%%%%%

ode_run(p,'steady'); % saves matfile with steady state model values

%% pregnancy simulations
% Make sure to 'turn on' female params above

load('steady');
Initial = Y(end,:); % use concentrations from earlier simulations
ode_run(p,'pregnancy'); % saves matfile with values over pregnancy

%% menstrual cycle simulations
% Make sure to 'turn on' female params above

[td,e2d] = Mean_e2d;
p.e2d = e2d; p.td = td;

ode_run(p,'menstrual'); % saves matfile with values over menstrual cycle

%% menstrual cycle plots

load('menstrual');
figure('Position',[10 10 900 600]);
subplot(2,2,1);
plot(T,met/met(1),T,sam/sam(1),'LineWidth',2);
hold on;
plot(T,sah/sah(1),T,hcy/hcy(1),'LineWidth',2);
hold on;
plot(T,(sam./sah)/(sam(1)/sah(1)),'k-.','LineWidth',2);
legend('Met','SAM','SAH','Hcy','SAM/SAH','FontSize',14,'Location','southeast'
);
xlim([0 28]);
ylabel('Relative level','FontSize',18);
xlabel('Day of menstrual cycle','FontSize',18);
ax = gca; ax.FontSize = 14;

subplot(2,2,2);
plot(T,vvms/vvms(1),T,vcbs/vcbs(1),'LineWidth',2);
hold on;
plot(T,vamd1/vamd1(1),'LineWidth',2);
hold on;
plot(T,frac/frac(1),'LineWidth',2);
legend('{\it V}_{MS}','{\it V}_{CBS}','{\it
V}_{AMD1}','frac','FontSize',14,'Location','southeast');
xlim([0 28]);
ylabel('Relative level','FontSize',18);
```

```
xlabel('Day of menstrual cycle','FontSize',18);
ax = gca; ax.FontSize = 14;

subplot(2,2,3);
plot(T,vgnmt/vgnmt(1),T,vgamt/vgamt(1),'LineWidth',2);
hold on;
plot(T,vdnmt/vdnmt(1),T,vpemt/vpemt(1),'LineWidth',2);
hold on;
legend('{\it V}_{GNMT}','{\it V}_{GAMT}','{\it V}_{DNMT}','{\it
V}_{PEMT}','FontSize',14,'Location','southeast');
xlim([0 28]);
ylabel('Relative level','FontSize',18);
xlabel('Day of menstrual cycle','FontSize',18);
ax = gca; ax.FontSize = 14;

subplot(2,2,4);
plot(T,mthf/mthf(1),T,vts/vts(1),'LineWidth',2);
hold on;
plot(T,vart/vart(1),T,glu/glu(1),'LineWidth',2);
hold on;
legend('5mTHF','{\it V}_{TS}','{\it
V}_{AICART}','GSH','FontSize',14,'Location','southeast');
xlim([0 28]);
ylabel('Relative level','FontSize',18);
xlabel('Day of menstrual cycle','FontSize',18);
ax = gca; ax.FontSize = 14;

%% run simulations for predefined parameter structure p

function ode_run(p,type)
% saves model concentrations and velocities in a matfile

GARP = 10;
AICARP = 2.1;
NADPH = 50;
SER = 468;
HCOOH = 500;
DUMP = 20;
GLY = 1850;
GAA = 10;
PE = 100;

pf = p.val(p.pf);
b12 = p.val(p.b12); % VVMS factor for b12 deficiency
b6 = p.val(p.b6);
f = p.val(p.f); %folate

% methylation Vmaxes with male-female differences
gnmtv = p.val(p.gnmtv);
othv = p.val(p.othv);
dnmtv = p.val(p.dnmtv);
gamtv = p.val(p.gamtv);
pemtv = p.val(p.pemtv);

if contains(type,'menstrual')
    ode_opt = {'RelTol',1e-6,'AbsTol',1e-8};
    dt = 0.01;
    TIME = 0:dt:28*20;
    % estradiol data vectors
```

```
    td = p.td;
    e2d = p.e2d;
end

if contains(type,'pregnancy')
    ode_opt = {'RelTol',1e-6,'AbsTol',1e-8};
    TIME = [0 290];
end

if contains(type,'steady')
    ode_opt = {};
    TIME = [0 1000];
    % estradiol effect on PEMT and CBS
    e2 = p.val(p.e2);
    ppe = PEMTspline(e2);
    ce = CBSspline(e2);
end

metin = p.val(p.METIN);
Sphmy = 10*(1+pf*((1.3)-1));

Initial = [(f)*4.32 (f)*6.85 (f)*0.02 (f)*0.77 (f)*0.99 (f)*5.22  ...
    17.68  29.01   3.34   1.79 0.0006 0.2692 0.7268 0 315.62 327.08  ...
    59.81  20.60 4.7];
[T,Y] = ode15s(@(t,y)msc(t,y,p,type),TIME,Initial,ode_opt);

if contains(type,'pregnancy') || contains(type,'menstrual')

    if contains(type,'pregnancy')
        e2 = E2pregnancy(T); e2 = e2';
    else
        T = 0:dt:28;
        Y = Y(end-28/dt:end,:);
        e2 = E2menstrual(T,td,e2d); e2 = e2';
    end

    % estradiol effect on PEMT and CBS
    ppe = PEMTspline(e2);
    ce = CBSspline(e2);
    % save time series
    mthf = Y(:,1);
    thf  = Y(:,2);
    fthf = Y(:,6);
    dhf  = Y(:,3);
    ch2 = Y(:,4);
    ch  = Y(:,5);
    met = Y(:,7);
    sam = Y(:,8);
    sah = Y(:,9);
    hcy = Y(:,10);
    gnmt = Y(:,11);
    gnmt5mthf = Y(:,12);
    mthfgnmt5mthf = Y(:,13);
    folatefree = 20*f;
    bet = Y(:,15);
    betald = Y(:,16);
    cho = Y(:,17);
    pc = Y(:,18);
    glu = Y(:,19);
```

```
    vdhfr = VDHFR(dhf,NADPH,e2);
    vts = VTS(DUMP,ch2,e2);
    vmhd = VMHD(ch2,ch);
    vmch = VMCH(ch,fthf);
    vpgt = VPGT(fthf,GARP);
    vart = VART(fthf,AICARP);
    vfts = VFTS(thf,HCOOH,fthf);
    vftd = VFTD(fthf);
    vch2 = VCH2(thf,HCOOH,ch2);
    vshmt = VSHMT(SER,thf,GLY,ch2,pf,b6);
    vmthfr = VMTHFR(ch2,NADPH,sam,sah,pf);
    vvms = VVMS(mthf,hcy,pf,b12);
    vbhmt = VBHMT(hcy,bet,sam,sah,pf);
    vah = VAH(sah,hcy);
    vcbs = VCBS(hcy,sam,sah,bet,b6,ce);
    vdnmt = VDNMT(sam,sah,dnmtv);
    vgnmt = VGNMT(sam,sah,mthf, GLY,gnmt,gnmt5mthf,gnmtv);
    vgamt = VGAMT(sam,sah,GAA,gamtv);
    vpemt = VPEMT(sam,sah,PE,ppe,pemtv);
    vbah = VBAH(betald);
    vcho = VCHO(cho);
    vppl = VPPL(pc);
    vsms =  VSMS(Sphmy);
    voth = VOTH(sam,sah,othv);
    vmatI = VMATI(met,sam);
    vmatIII = VMATIII(met,sam);
    frac = vcbs./(vcbs+ vvms + vbhmt);
    vasmt1 = VASMT1(sam,sah);
    vamd1 = VAMD1(sam);
    folatesum = mthf+thf+dhf+ch2+ch+fthf + gnmt5mthf + 2*mthfgnmt5mthf;
    sixsum = mthf+thf+dhf+ch2+ch+fthf;
    transsulf = vcbs;
    transmeth = vgnmt+vdnmt+vgamt+vpemt+voth;
    remeth = vvms+vbhmt;
    if contains(type,'menstrual')
        save(['menstrual']);
    else
        save(['pregnancy']);
    end
end

if contains(type,'steady')
    % save the final concentrations and rates
    mthf = Y(length(T),1);
    thf  = Y(length(T),2);
    fthf = Y(length(T),6);
    dhf  = Y(length(T),3);
    ch2 = Y(length(T),4);
    ch  = Y(length(T),5);
    met = Y(length(T),7);
    sam = Y(length(T),8);
    sah = Y(length(T),9);
    hcy = Y(length(T),10);
    gnmt = Y(length(T),11);
    gnmt5mthf = Y(length(T),12);
    mthfgnmt5mthf = Y(length(T),13);
    folatefree = 20*f;
    bet = Y(length(T),15);
```

```
    betald = Y(length(T),16);
    cho = Y(length(T),17);
    pc = Y(length(T),18);
    glu = Y(length(T),19);
    vdhfr = VDHFR(dhf,NADPH,e2);
    vts = VTS(DUMP,ch2,e2);
    vmhd = VMHD(ch2,ch);
    vmch = VMCH(ch,fthf);
    vpgt = VPGT(fthf,GARP);
    vart = VART(fthf,AICARP);
    vfts = VFTS(thf,HCOOH,fthf);
    vftd = VFTD(fthf);
    vch2 = VCH2(thf,HCOOH,ch2);
    vshmt = VSHMT(SER,thf,GLY,ch2,pf,b6);
    vmthfr = VMTHFR(ch2,NADPH,sam,sah,pf);
    vvms = VVMS(mthf,hcy,pf,b12);
    vbhmt = VBHMT(hcy,bet,sam,sah,pf);
    vah = VAH(sah,hcy);
    vcbs = VCBS(hcy,sam,sah,bet,b6,ce);
    vdnmt = VDNMT(sam,sah,dnmtv);
    vgnmt = VGNMT(sam,sah,mthf, GLY,gnmt,gnmt5mthf,gnmtv);
    vgamt = VGAMT(sam,sah,GAA,gamtv);
    vpemt = VPEMT(sam,sah,PE,ppe,pemtv);
    vbah = VBAH(betald);
    vcho = VCHO(cho);
    vppl = VPPL(pc);
    vsms =  VSMS(Sphmy);
    voth = VOTH(sam,sah,othv);
    vmatI = VMATI(met,sam);
    vmatIII = VMATIII(met,sam);
    frac = vcbs/(vcbs+ vvms + vbhmt);
    vasmt1 = VASMT1(sam,sah);
    vamd1 = VAMD1(sam);
    folatesum = mthf+thf+dhf+ch2+ch+fthf + gnmt5mthf + 2*mthfgnmt5mthf;
    sixsum = mthf+thf+dhf+ch2+ch+fthf;
    transsulf = vcbs;
    transmeth = vgnmt+vdnmt+vgamt+vpemt+voth;
    remeth = vvms+vbhmt;
    save(['steady']);
end
end

%% utility functions used in parameter setup

function p = parameters

p.pf = 1;
p.b12 = 2;
p.b6 = 3;
p.f = 4;
p.METIN = 5;
p.BETIN = 6;
p.e2 = 7;
p.gnmtv = 8;
p.othv = 9;
p.dnmtv = 10;
p.gamtv = 11;
p.pemtv = 12;
```

```
p.val(p.pf) = 0; % percent female values for VBHMT,VSHMT,VMS,VMTHFR,Sphmy
                 % with 0=male and 1=female
p.val(p.b12) = 1;
p.val(p.b6) = 1;
p.val(p.f) = 1; % normal folate = 1
p.val(p.METIN) = 40;
p.val(p.BETIN) = 13;
p.val(p.e2) = 0.09;

% methylation Vmaxes for standard male
p.val(p.gnmtv) = 0.3;
p.val(p.othv) = 1.4;
p.val(p.dnmtv) = 2;
p.val(p.gamtv) = 1.6;
p.val(p.pemtv) = 0.6;

end

function pemtActivity = PEMTspline(e2)
%{
Input:
    e2              estradiol
Output:
    pemtActivity  PEMT enzyme activity
%}

pemtActivity = 1 + 1.2904*e2./(1.0402+e2);

end

function cbsActivity = CBSspline(e2)
%{
Input:
    e2              estradiol
Output:
    cbsActivity   CBS enzyme activity
%}

cbsActivity = 1 + (e2-.09)./(.67+(e2-.09));

end

function e2 = E2menstrual(t,td,e2d)
%{
Input:
    td              day of menstrual cycle data
    e2d             concentration of estradiol in nmol/L
Output:
    e2              estradiol at day t of menstrual cycle
%}

pd = spline(td,e2d);
ed = @(t) ppval(pd,t); % estradiol vs time

e2 = ed(mod(t,28));

end

function e2 = E2pregnancy(t)
```

```
% estradiol at day t of pregnancy

[td,e2d] = pregnancydata;
pd = spline(td,e2d);
ed = @(t) ppval(pd,t); % estradiol vs time
e2 = ed(t);

end

%% RHS of ordinary differential equations

function dy = msc(t,y,p,type)

dy=zeros(19,1);

GLY = 1850;
GAA = 10;
k1 = 50;  % k1,k2,k3,k4 based on Kd values from Luka08 (and wagner)
k2 = 1;
k3 = 1;
k4 = 1.6;
n = 1;    % normal niacin = 1
PE = 100;
cholin = 200;

% percent female values for VBHMT,VSHMT,VMS,VMTHFR,Sphmy
% with 0=male and 1=female
pf = p.val(p.pf);

b12 = p.val(p.b12); % VVMS factor for b12 deficiency
b6 = p.val(p.b6); % factor for b6
f = p.val(p.f); % folate (normal=1)

betin = p.val(p.BETIN);
Sphmy = 10*(1+pf*((1.3)-1)); % (female=13)

METIN = p.val(p.METIN);

% methylation Vmaxes with male-female differences
gnmtv = p.val(p.gnmtv);
othv = p.val(p.othv);
dnmtv = p.val(p.dnmtv);
gamtv = p.val(p.gamtv);
pemtv = p.val(p.pemtv);

%%%%%%%%%%
if contains(type,'menstrual')
    % estradiol data vectors
    td = p.td;
    e2d = p.e2d;
    %e2 during menstrual cycle
    e2 = E2menstrual(t,td,e2d);
end

if contains(type,'pregnancy')
    %e2 during pregnancy
    e2 = E2pregnancy(t);
end

if contains(type,'steady')
```

```
    e2 = p.val(p.e2);
end

% estradiol effect on PEMT and CBS
ppe = PEMTspline(e2);
ce = CBSspline(e2);
%%%%%%%%%%

dy(1) =  n.*VMTHFR(y(4),50,y(8),y(9),pf) - VVMS(y(1),y(10),pf,b12) +
k2.*y(12) - 2.*k1.*y(1).*y(11) + k4.*y(13) - k3.*y(1).*y(12);
dy(2) =  VFTD(y(6)) +  VVMS(y(1),y(10),pf,b12) + VPGT(y(6),10) +
VART(y(6),2.1) - VFTS(y(2),900,y(6)) - VSHMT(468, y(2), 1850,y(4),pf,b6) -
VCH2(y(2),500,y(4))  + n.*VDHFR(y(3),50,e2);
dy(3) =  VTS(20, y(4),e2) - n.*VDHFR(y(3), 50,e2);
dy(4) =  VSHMT(468, y(2), 1850,y(4),pf,b6)  +  VCH2(y(2),500,y(4))  -
VTS(20, y(4),e2) - n.*VMTHFR(y(4),50,y(8),y(9),pf) - n.*VMHD(y(4),y(5));
dy(5) =  n.*VMHD(y(4),y(5)) - VMCH(y(5),y(6));
dy(6) =  VMCH(y(5),y(6)) + VFTS(y(2),900,y(6)) -  VPGT(y(6),10) -
VART(y(6),2.1) - VFTD(y(6));
dy(7) =  VBHMT(y(10),y(15),y(8),y(9),pf) + VVMS(y(1),y(10),pf,b12) +
metin(t,METIN) - VMATI(y(7),y(8)) -  VMATIII(y(7),y(8));
dy(8) =  VMATI(y(7),y(8)) +  VMATIII(y(7),y(8)) -
VGNMT(y(8),y(9),y(1),GLY,y(11),y(12),gnmtv) - VDNMT(y(8),y(9),dnmtv) -
gab(t).*VGAMT(y(8),y(9),GAA,gamtv) - VPEMT(y(8),y(9),PE,ppe,pemtv) -
VOTH(y(8),y(9),othv) - VAMD1(y(8));
dy(9) =  VGNMT(y(8),y(9),y(1), GLY,y(11),y(12),gnmtv) +
VDNMT(y(8),y(9),dnmtv) + gab(t).*VGAMT(y(8),y(9),GAA,gamtv)+
VPEMT(y(8),y(9),PE,ppe,pemtv) + VOTH(y(8),y(9),othv) - VAH(y(9),y(10));
dy(10) = VAH(y(9),y(10)) - VCBS(y(10),y(8),y(9),y(15),b6,ce) -
VBHMT(y(10),y(15),y(8),y(9),pf) - VVMS(y(1),y(10),pf,b12);
dy(11) = k2.*y(12) - 2.*k1.*y(1).*y(11);
dy(12) = -k2.*y(12) + 2.*k1.*y(1).*y(11) - k3.*y(1).*y(12) + k4.*y(13);
dy(13) = k3.*y(1).*y(12) - k4.*y(13);
dy(14) = sin(t);
dy(15) = betin + VBAH(y(16)) - VBHMT(y(10),y(15),y(8),y(9),pf) -.0096*y(15);
dy(16) = VCHO(y(17)) - VBAH(y(16)) - .01*y(16);
dy(17) = cholin + VPPL(y(18)) - VCHO(y(17)) - cholout(y(17));
dy(18) = VPEMT(y(8),y(9),PE,ppe,pemtv) + VSMS(Sphmy) - VPPL(y(18));
dy(19) = VCBS(y(10),y(8),y(9),y(15),b6,ce) - .015*y(19);

% y(1) = 5mTHF
% y(2) = THF
% y(3) = DHF
% y(4) = 510CH2
% y(5) = 510CH
% y(6) = 10fTHF
% y(7) = met
% y(8) = sam
% y(9) = sah
% y(10) = hcy
% y(11) = GNMT
% y(12) = GNMT-5mTHF
% y(13) = 5mTHF-GNMT-5mTHF
% y(15) = betaine
% y(16) = bet-ald
% y(17) = cho
% y(18) = pc   Phosphocholine

end
```

```
%% experimental data

function [td,e2d] = Mean_e2d
% mean E2 data throughout menstrual cycle

td = 0:1:28;
e2d = [0.1368;0.1335;0.1416;0.1498;0.1775;0.1807;0.2378;...
    0.2573;0.3143;0.3795;0.6075;0.8112;1.1533;0.9268;0.5000;...
    0.2948;0.4137;0.4870;0.5750;0.5310;0.5961;0.6466;0.6271;...
    0.5831;0.4626;0.3974;0.3078;0.2035;0.1351];

end

function [td,e2d] = pregnancydata

td = [0;21;42;59.4924078091106;76.9848156182210;89.5574837310195;...
    104.863340563991;117.162689804773;133.015184381779;...
    161.167028199566;175.379609544469;189.045553145336;...
    203.258134490239;217.197396963123;231.136659436009;...
    246.715835140998;259.015184381779;274.594360086768;290.173535791757];
e2d = [0.4368;2.3760;4.3153;...
    5.93062615384600;7.06026923076930;11.0140200000000;...
    13.5557169230770;16.5210300000000;31.9124169230770;...
    34.5953192307690;38.1254538461540;34.7365246153850;...
    38.9726861538460;40.2435346153850;49.2806792307690;...
    53.0932246153850;54.6464838461540;56.4821538461540;58.4590292307690];

end

%% reaction velocities

function a = VAH(b,c)
%b = adohcy concentration
%c = hcy concentration
    k20 = (1.4)*320;
    k21 = 6.5;
    k22 = 150;
    k200 = 755;
    a = (1)*k20.*(b./k21)./(1 + (b./k21)) - (6)*k200.*(c./k22)./(1 +
(c./k22));
end

function a = VAMD1(b)
%b = sam
    Km = 245;
    Vmax = 100;
    a = Vmax.*b./(Km+b);
end

function a = VART(b,c)
%b = 10fTHF
%c = AICARP (needs to be specified in main program)
    k1 =   5.9;  %Km10fTHF
    k2 =    100 ;   %KmAICARP
    V  =   (1)*45000 ;
    a = V.*(b./k1).*(c./k2)./(1 + (b./k1) + (c./k2) +  (b./k1).*(c./k2));
end
```

```
function a = VASMT1(b,c)
%b = SAM
%c = SAH
    k1 = 5;    %Vmax
    k2 = 50 ;  %Km for SAM from Song10
    k5 = 10;  %inhibition by SAH
    a = k1.*b./((k2.*(1+c./k5) + b));
end

function a = VASMT2(b,c,d,e)
%b = SAM, c = SAH, d = LiAs, e = LMMA
    k1 = 200;  %Vmax
    k2 = 50 ;  %Km for SAM from Song10
    k3 =  1  ; %Ki of SAH
    k4 = 4.6;  %Km for iAs
    k5 = 1.26; %inhibition by iAS
    k6 = 40;   %Ki for LMMA
    a = k1.*e.*b./((k4 + e).*(k2 + b).*(1+d./k5));
end

function a = VBAH(b)
%b = betaine aldehyde
    k17 = 45;
    k18 = 250;  %Km for betaine aldehyde
    a =  k17.*b./(k18 + b);
end

function a = VBHMT(h,b,d,c,p)
% b = betaine, h = hcy, d = adomet, c = adohcy
% p = percent female adaptation
    k17 = (1+p*((0.6)-1))*2000; %female=0.6
    k18 = 12;   %Km for Hcy %Finkelstein90,Garrow06
    k19 = 2000; %Garrow06
    %NORMAL
    a =  (.8)*(k17.*h.*b./((k18+h).*(k19+b))).*(exp(-.0021.*(c + d))).*...
        (exp(+.0021.*(32.3))).*(1 + (.16).*(b-315)/315); %betaine effect
end

function a = VCBS(b,d,e,f,b6,fe)
% b= hcy, d = adomet, e = adoHcy, f = betaine
% p = percent female adaptation (male=0,female=1)
% fe = cbs fold dependent on e2
    k30 = fe*b6*29*170/4.2;
    k31 = 170;
    a = (.75).*k30.*b./(k31+b) .* (4.6).*(d+e)./(14+(d+e))...
        .*(1 + (1).*H1(f-315).*(.25).*(f-315)./(100 + (f-315)));
        % effect of betaine suppl.; this term is 1 in male model
end

function a = VCH2(b,c,d)
% b = THF
% c = HCOOH (must be specified in main program)
% d = 510CH2
    k1 = 0.15;
    k2  = 12;
    a = k1.*b.*c - k2.*d;
end

function a = VCHO(b)
```

```
%b = choline
    k17 = 125;
    k18 = 200;  %Km for choline
    a = k17.*b./(k18 + b);
end

function a = VDHFR(b,c,e2)
% b = DHF
% c = NADPH  (needs to be specified in main program)
% e2 = estradiol
    k1 = 0.5;
    k2 = 4.0;
    V = (100)*50;
    a = (1+2*(e2)./(19+(e2)))*V.*(b./k1).*...
        (c./k2)./(1 + (b./k1) + (c./k2) +  (b./k1).*(c./k2));
end

function a = VDNMT(b,c,dnmtv)
%b = adomet
%c = adohcy
    k66 = (dnmtv)*(2.5);
    k77 = 1.4;
    k78 = 1.4;
    a =  k66.*b./(k77.*(1 + c./k78) + b);
end

function a = VFTD(b)
% b = 10fTHF
    k1 = 20;
    V = (1)*4*3500;
    a = V.*(b./k1)./(1 + (b./k1));
end

function a = VFTS(b,c,d)
% b = THF
% c =  HCOOH  (needs to be specified in main program)
% d = 10fTHF
    k1 = 3;
    k2 = 43;    %KmHCOOH
    V  = (1)*2000;
    a = V.*(b./k1).*(c./k2)./(1 + (b./k1) + (c./k2) + (b./k1).*(c./k2));
end

function a = VGAMT(b,c,d,gamtv)
%b = SAM
%c = SAH
    k1 = (gamtv).*(90);    %Vmax
    k2 = 49;    % Km for SAM   HCY book
    k3 = 16;    % Ki for SAH   HCY book
    k4 = 13.3;  % Km for GAA   dasilva09
    a = k1.*(b./(k2.*(1 + c./k3) + b));
end

function a = VGNMT(b,c,d,e,g,fg,gnmtv)
%b = sam, c = sah, d = 5mTHF, e = glycine
%g = gnmt, fg = gnmt-5mTHF
    k2 = 100;    %Km for SAM
    k3 = 35;     %Ki for SAH
    k4 = 12.2;   %Km glycine
```

```
    k1 = (gnmtv)*(1750);
    a = k1.*(g + (.5).*fg).*(b./((k2.*(1 + c./35)) + b));
end

function a = VMATI(b,c)
%c = adomet, b = met
    k1 = 260;
    k2 = 41;
    a = (1)*(0.23 + 0.8.*exp(-0.0026.*c)).*k1.*(b./k2)./(1 + (b./k2));
end

function a = VMATIII(b,c)
%c = adomet, b = met
    k1 = 220;
    k2 = 300;
    k3 = 360*360; %VMATIi (inhib for adomet)
    a = k1.*(b.^(1.21)./(k2 + b.^(1.21)).*(1 +7.2.*c.^(2)./(k3 + c.^(2))));
end

function a = VMCH(b,c)
% b = 510CH
% c = 10fTHF
    k1 = 250;
    V1 = (1)*800000;
    k2 = 100;
    V2 = (1)*20000;
    a =  V1.*(b./k1)./(1 + b./k1) - V2.*(c./k2)./(1 + c./k2);
end

function a = VMETH(b,c)
%b = adomet
%c = adohcy
    k6 = 2400;
    k7 =  10.0;
    k66 = (1)*180;
    k77 = 1.4;
    k78 = (.6)*1.4;
    a =  k66.*b./(k77.*(1 + c./k78) + b);
end

function a = VMHD(b,c)
%   b = 510CH2
%   c = 510CH
    k1 = 2;
    V1 = (1)*200000;
    k2 = 10;
    V2 = (1)*594000;
    a =  V1.*(b./k1)./(1 + b./k1)  - V2.*(c./k2)./(1 + c./k2);
end

function a = VMTHFR(b,c,d,e,p)
% b = 510CH2
% c = NADPH (specified in main program)
% d = adomet
% e = adohcy
% p = percent female adaptation
    k1 = 50;
    k2 = 16;
    V = (1+p*((0.8)-1))*2000;  %female=0.8
```

```
    a = (V.*b.*c./((k1+b).*(k2+c))).*(10./(10 + (d-e))).*((10 + 25.7)./10);
end

function a = VOTH(b,c,othv)
%b = SAM
%c = SAH
    k1 = (othv)*(40);
    k2 = 50;
    k3 = 15;
    a = k1.*b./(k2.*(1 + c./k3) + b);
end

function a = VPEMT(b,c,d,fe,pemtv)
%b = sam
%c = sah
%d = pe
% p = percent female adaptation
% fe = pemt fold dependent on e2
    k1 = pemtv*fe*98;   %Vmax
    k2 = 18.2;  % Km for SAM
    k3 = 3.8;   %Ki for SAH
    k4 = 5000;  %Km for PE
    d=100;
    a = k1.*((b./(k2 + b))).*(1./(1 + c./k3)).*(25*d/(k4+d));
end

function a = VPGT(b,c)
% b = 10fTHF
% c =  GARP  (needs to be specified in main program)
    k1 = 4.9;  %Km10fTHF
    k2 = 520;   %KmGARP
    V  = (1)*16200;
    a = V.*(b./k1).*(c./k2)./(1 + (b./k1) + (c./k2) + (b./k1).*(c./k2));
end

function a = VPPL(b)
%b = pc
    k17 = 525;
    k18 = 400;  %Km for phosphocholine
    a =  k17.*b./(k18 + b);
end

function a = VSHMT(b,c,d,e,p,b6)
% b = Serine  (specified in main program)
% c = THF
% d = Glycine (specified in main program)
% e = 510CH2
% f = free SHMT
% positive direction from THF towards 510CH2
% p = percent female adaptation
    k1  = 600;  %Km for ser
    k2  = 50;   %Km for THF
    V1 = b6*(1+p*((2.2)-1))*40000;  %female=2.2
    k3 = 3000;  %Km for gly
    k4 = 3200;  %Km for ch2
    V2 = b6*(1+p*((2.2)-1))*2500000;  %female=2.2
    a = V1.*(b./k1).*(c./k2)./(1+(b./k1)+(c./k2)+(b./k1).*(c./k2))...
        -V2.*(d./k3).*(e./k4)/(1+(d./k3)+(e./k4)+(d./k3).*(e./k4));
end
```

```
function a = VSMS(b)
%b = sphmy
    k17 = 30;   %30
    k18 = 20;  %Km for phosphocholine
    a =  k17.*b./(k18 + b);
end

function a = VTS(b,c,e2)
% b = DUMP (needs to be specified in main program)
% c =  510CH2
% e2 = estradiol
    k1 =   6.3 ;  %KmDUMP
    k2 =   14;   %Km510CH2
    V  = (1)*(100)*50;
    a = (1+2*(e2)./(19+(e2)))*V.*(b./k1).*...
        (c./k2)./(1 + (b./k1) + (c./k2) +  (b./k1).*(c./k2));
end

function a = VVMS(c,b,p,b12)
% b = hcy
% c = 5mTHF
% p = percent female adaptation
% b12 = effect of b12 deficiency
    k12 = b12*(1)*(1+p*((1.35)-1))*(.5)*(1.2)*406; %female=1.35
    k13 = 25;   %K for mTH4 (um)
    k14 =  1;
    a = (1)*k12.*((b./k14)./(1 + (b./k14))).*(c./k13)./(1 + (c./k13));
end

%c = choline
function a = cholout(c)
    k1 = (.97)*220;   %Vmax
    k2 = 5;  % Km
    a = k1.*(c./(k2 + c));
end

function f = gab(t)
    n=length(t);
    for i=1:n
      if t(i) < 1000
        f(i) = 1;
      else f(i) = 1;
      end
    end
end

function f = H1(a)
    if a < 0
          f = 0;
    else
        f = 1;
    end
end

function f=metin(t,p)
% to experiment with metin over time
    f = p;
end
```
